# Composition Based Oxidation State Prediction of Materials Using Deep Learning Language Models

**DOI:** 10.1002/advs.202301011

**Published:** 2023-08-07

**Authors:** Nihang Fu, Jeffrey Hu, Ying Feng, Gregory Morrison, Hans‐Conrad zur Loye, Jianjun Hu

**Affiliations:** ^1^ Department of Computer Science and Engineering University of South Carolina Columbia SC 29201 USA; ^2^ Dutch Fork High School Irmo SC 29063 USA; ^3^ Hangzhou University of Electronic Science and Technology Hangzhou 311305 China; ^4^ Department of Chemistry and Biochemistry University of South Carolina Columbia SC 29201 USA

**Keywords:** deep learning, language model, material discovery, material screening, neural networks, oxidation states, transformer

## Abstract

Oxidation states (OS) are the charges on atoms due to electrons gained or lost upon applying an ionic approximation to their bonds. As a fundamental property, OS has been widely used in charge‐neutrality verification, crystal structure determination, and reaction estimation. Currently, only heuristic rules exist for guessing the oxidation states of a given compound with many exceptions. Recent work has developed machine learning models based on heuristic structural features for predicting the oxidation states of metal ions. However, composition‐based oxidation state prediction still remains elusive so far, which has significant implications for the discovery of new materials for which the structures have not been determined. This work proposes a novel deep learning‐based BERT transformer language model BERTOS for predicting the oxidation states for all elements of inorganic compounds given only their chemical composition. This model achieves 96.82% accuracy for all‐element oxidation states prediction benchmarked on the cleaned ICSD dataset and achieves 97.61% accuracy for oxide materials. It is also demonstrated how it can be used to conduct large‐scale screening of hypothetical material compositions for materials discovery.

## Graphical abstract

Deep learning neural language model is used to learn the relationships of materials compositions and elemental oxidation states and exploit this deep dark knowledge for accurate prediction of atomic site oxidation states for materials given only their compositions. This model can be used to speed up high‐throughput generative discovery of new materials.

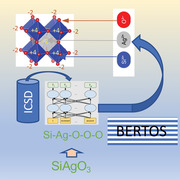

## 1 Introduction

Oxidation states (OS) are the charges on atoms due to electrons gained or lost upon applying ionic approximation to their bonds, and represent the fundamental attributes of elements that help to explain redox reactions, reactivity, chemical bonding, and chemical properties of different elements and compounds.^[^ ^1^, ^2^ ^]^ In electrochemistry, oxidation states are used to represent relevant compounds and ions in Latimer and Frost diagrams, and they can also be used for determining the charge neutrality of chemical compounds to screen potential hypothetical materials generated by computational design algorithms.^[^ ^3^ ^]^ Identifying the correct oxidation states is essential, for example for the study of transition metal complexes.^[^ ^4^ ^]^ In material science, oxidation states are useful in determining why one compound might be more suitable than another and have also been used in structure prediction for materials discovery by chemical analogy.^[^ ^5^ ^]^ Another major usage of oxidation state prediction is to check the chemical validity of hypothetical material compositions or structures. In recently emerged high‐throughput material discovery paradigm, deep learning based generative models have been developed for large‐scale generation of hypothetical material compositions,^[^ ^6^, ^7^ ^]^ which need to be filtered in terms of their chemical validity and sythesizability using automated methods. In this scenario, there is no structural information available and the composition based oxidation state prediction model can greatly narrow down the search of new materials.

Chemists determine the elemental oxidation states of a compound via numerous approaches, including the extrapolation of a chemical bond's polarity (from electronegativity differences, dipole moments, or quantum‐mechanical calculations of charges) or assigning electrons based on the number of electrons contributing to a molecular orbit. Commonly used rules for assigning oxidation states in chemical equations and for writing chemical formulas are as follows: the cations (positively charged ions) are listed first, followed by the anions. The oxidation state of a free element is 0. Assigning formal oxidation states, which represent the hypothetical charge that an atom would have when all the electrons in an atom's bonds are allocated to the higher electronegative element, are detailed in many chemistry textbooks. Once assigned, the sum of the oxidation states of atoms in neutral compounds is always 0, and the sum of oxidation states in polyatomic ions is equal to the charge of the ion. Assigning formal oxidation states results in an oxidation state hydrogen of typically +1, of ‐2 for oxygen (except in peroxides or oxygen fluorine complexes), of +1 and +2 respectively for Group 1 and 2 cations, and typically of +1 or +3 for Group 13 cations. The challenge of assigning oxidation states in general lies in the fact that many elements are able to take on multiple oxidation states that vary depending on their local chemical environment and overall complex or material composition, confirming that most rules are only applicable to specific situations and exhibit several different types of exceptions. To address this challenge, recently machine learning‐based algorithms have been proposed for oxidation state assignments for metal atoms in compounds.^[^ ^8^, ^9^, ^10^, ^11^ ^]^ Jablonka et al.^[^ ^8^ ^]^ proposed the use of a machine learning model, trained with data from the Cambridge Structural Database (CSD), to automatically assign oxidation states to the metal ions in metal–organic frameworks. They found that charge‐partitioning based computational techniques are unable to remove ambiguity, and therefore, they proposed applying of collective knowledge from chemists to assign oxidation states instead of using the rule‐based deduction method based on formal counting rules. In their approach, they parsed the OS of the metal atoms from the compound names of Metal‐Organic Framework (MOF) structures in the CSD, encoded the local chemical numerically, and trained a group of machine learning (ML) models. The models then made predictions based “vote” between four base models to classify the oxidation states. Amin et al.^[^ ^9^ ^]^ used supervised and unsupervised machine learning for predicting the oxidation states of Mn ions in the oxygen‐evolving complex of photosystem II by predicting the S‐state of the X‐ray, XFEL, and CryoEM structures. They trained a decision tree classifier and K‐means clustering models using Mn compounds from the Cambridge Crystallographic Database. The model uses two types of features: (1) the average bond length between the Mn and equatorial ligands and (2) the average bond length between the Mn and the axial ligands. The model was validated by using three different methods: predicting the Mn oxidation states in other protein structures with higher resolution, calculating the spin densities of the Mn in two of the mismatched structures of different S‐states resolved by different groups, and validating the predictions by comparing them against the valence bond model. Shevchenko et al.^[^ ^10^ ^]^ designed a random forest (RF) algorithm to predict the oxidation states of atoms and topological features of crystal structures using a predictive scheme for oxidation states in three stages: (1) selecting features and considering complex correlations between them, (2) training the RF model and choosing a set of hyperparameters, and (3) verifying the scheme with the testing samples by predicting oxidation states values and finding quality criteria. They selected features by evaluating relations between oxidation states and other descriptors with Pearson and Spearman correlations. The features were required to be strongly correlated with OS. Automated oxidation‐state assignment for metal sites in coordination complexes has also been studied in Ref. [11], and in this work, Reeves et al. presented an automated workflow for oxidation‐state assignment in transition‐metal coordination complexes using the Cambridge Structural Database (CSD) Python API (application programming interface) scripts that complements the bond valence sum method for improved assignment confidence. Oxidation states of binary oxides from data analytics of the electronic structure were also investigated by Posysaev et al.,^[^ ^12^ ^]^ who used a simple machine learning algorithm with linear regression to show that a correlation exists for the binary oxide systems.

So far, all machine learning‐based oxidation state prediction models are based on local structures of metal ions. There is no study on composition‐based ML models for OS prediction and there are no ML models for predicting the OS of nonmetal elements with the structural information. In Pymatgen,^[^ ^13^ ^]^ there exists an oxidation state guessing function oxide_state_guess, which is based on an exhaustive enumeration algorithm and can give multiple possible charge‐neutral oxidation state assignments. Currently, for composition‐based oxidation state assignments, this can only be done using some heuristic rules by human experts, which has no automated software available for large‐scale automated annotation of hypothetical candidate material compositions. In Ref. [5], Davies et al. analyzed the preferences of cations and anions to take preferred oxidation states and summarized some rules for assigning oxidation states for compositions, which are then used to narrow down possible compositions for materials discovery.

In this work, we propose a novel deep learning‐based BERT^[^ ^14^ ^]^ transformer language model (BERTOS) for composition‐based oxidation state assignment for inorganic compounds. Our work is inspired by the recent success of deep learning in learning protein sequence patterns,^[^ ^15^ ^]^ predicting protein structures,^[^ ^16^ ^]^ predicting protein properties,^[^ ^17^ ^]^ and even learning the structural patterns from protein sequences alone.^[^ ^18^ ^]^ Despite the difference between protein sequences and material formulas, recently, deep learning‐based generative models for composition design of inorganic material compositions^[^ ^6^, ^7^ ^]^ using generative adversarial networks^[^ ^3^ ^]^ and transformer language models^[^ ^19^ ^]^ were successfully developed, both of which are shown to be able to learn the chemical rules of material and molecule compositions.^[^ ^6^, ^7^, ^20^ ^]^ In these methods, the material compositions and molecules are represented as sequences of element symbol sequences, which are fed to the transformer network trained using the self‐supervised learning scheme. The trained language models were then used for the generative design of new candidate material compositions or molecules. In this work, we map the composition‐based oxidation state prediction problem as the token classification problem, in which the material formulas are converted into a sequence of element tokens sorted by their electronegativities and the output is the oxidation state of each atom. Our models are based on the BERT transformer language model and have demonstrated great performance for OS prediction.

## 2 Experimental Section

### 2.1 Dataset Preparation

The first step for oxidation state prediction modeling was to collect the datasets. The raw dataset was obtained from the inorganic crystal structures database (ICSD).^[^ ^21^ ^]^ Each structural cif file of ICSD database contains the composition and corresponding oxidation states of each atomic site. First, remove those formulas with fractional OS or with more than 200 atoms, and then remove all compounds to which only zero oxidation states were assigned (e.g., for most intermetallics). Since ICSD contains many cif structure files that neglect the hydrogen atoms, an algorithm was developed to add the hydrogen atoms back to the structure along with their OS. To deal with the polymorphic phases issue (one composition has multiple crystal phases), only one composition was kept with the most frequent oxidation assignment. If several OS assignments have equal frequency, the one with the maximum common oxidation states was picked.

In total, OS‐ICSD dataset was obtained with 52 147 samples. Further, extract only charge‐neutral compositions which do not contain fractional numbers of atoms to form the OS‐ICSD‐CN dataset (37 424 samples). Similarly, OS‐ICSD‐oxide dataset (35 886 samples) was obtained by picking only oxides from the OS‐ICSD dataset. Then, the OS‐ICSD‐CN‐oxide dataset (24 229 samples) was obtained from the OS‐ICSD‐CN dataset by selecting oxides formulas. For each of these four datasets, it was segmented into three subsets: training, validation, and test sets. The sample statistics are shown in Table S1 (Supporting Information) and the OS distributions for each element in these four datasets are shown in Table S2 (Supporting Information). Using this data partition approach, one guarantees that there are no overlaps between any pair of the training set and the test set. (See Supporting Information for more details).

### 2.2 The Transformer Neural Network Model for OS Prediction

**Figure 1:**
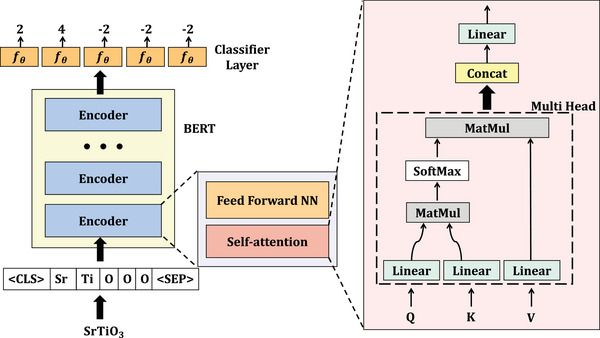
Architecture of BERTOS neural network model for oxidation state prediction. The overall framework (the left part) is composed of a BERT language model and a classifier layer *f*_θ_. Here, BERT consists of multiple stacked layers of encoders, and each encoder contains a self‐attention network and a feed‐forward network (the middle part). The rightmost part is the detailed multi‐head self‐attention network, which uses the query (Q), key (K), and value (V) mechanism to learn pair‐wise correlations among tokens at different positions within a given window.

The composition‐based oxidation state prediction problem as a token classification problem in natural language processing was formulated.^[^ ^22^ ^]^ The transformer language model^[^ ^23^ ^]^ framework was adopted for token classification as shown in **Figure** **1**. It contains two modules including a BERT neural network for learning the token representation and a dense network for the token classification. First, for the input, the regular material full formula/composition was expanded into a sequence of element symbol tokens. Using ternary compounds as an example, a typical material composition could be represented as *A*_*x*_ *B*_*y*_ *C*_*z*_ where A/B/C are elements and x/y/z were the number of atoms of corresponding elements. The same rule applies to compounds with a different number of elements. If one only considers the cases where x/y/z are integers, the formula could be expanded into *A*_1_ *A*_2_ … *A*_*x*_ *B*_1_ *B*_2_ … *B*_*y*_ *C*_1_ *C*_2_ … *C*_*z*_ where the elements are ordered by electronegativities. For example, *SrTiO*_3_ could be expanded to *Sr* *Ti* *O* *O* *O*, which becomes a regular sequence similar to a natural text sequence of words, a sequence of amino acids, or a SMILES representation of a molecule. With the expanded element sequence, the oxidation assignment problem for atoms becomes equivalent to the token classification in natural language understanding.^[^ ^24^ ^]^

The first module of the BERTOS framework was the Bidirectional Encoder Representations from Transformers (BERT) transformer language model. The BERT model was a transformer based neural network originally designed for natural language processing tasks. The core of BERT was a transformer model with a variable number of encoder layers and self‐attention heads (As shown in Figure 1). BERT models could be trained on two tasks: masked language modeling and next token prediction to learn contextual embeddings for tokens in the training process. Here, the BERT model was trained in a masked way. The raw representation of tokens was just a one‐hot encoding of elements without any additional elemental or structural properties. The goal of the BERT network was to learn the position‐aware encoding of each token within the formula sequence context (such as the oxidation‐states‐based charge‐neutrality constraints^[^ ^6^ ^]^). The network could be used to generate the elemental embedding representation for each element symbol in the formula.

The second module of the BERTOS framework was a shared regular feed‐forward fully connected neural network *f*_θ_ for mapping the BERT‐generated element embedding into their corresponding oxidation states. *f*_θ_ receives the embeddings from the BERT network to predict the oxidation states for the input tokens. This network was trained together with the BERT network with the supervised signal of actual oxidation states of atoms within the formulas. The details of the network structure parameters and the training hyper‐parameters are described in Supporting Information.

### 2.3 Roost Model Training

To evaluate the performance of the BERTOS model for screening hypothetical materials, the formation energy distribution of candidate material compositions filtered by the BERTOS model was compared. The Roost neural network model^[^ ^25^ ^]^ was used for formation energy per atom prediction of candidate formulas, which was trained with 100 133 non‐duplicate formulas from Materials Project, each formula picking their lowest formation energy per atom. The default network parameters were used with batch size 4096. The MAE error for the validation set was 0.108 eV. This model was one of the best models for composition based formation energy prediction.

### 2.4 Evaluation Criteria

Atomic site accuracy *P*_*S*_ measures the percentage of correctly predicted oxidation states of all atoms in all compositions of the test set.

(1) $$P_{S}=\frac{N_{atom}^{os}}{N_{atom}}$$

where $N_{atom}^{os}$ was the number of atoms with correct predicted oxidation states; *N*_*atom*_ was the total number of atoms in all compositions.

Element‐specific accuracy *P*_*E*_ measures the percentage of correctly predicted oxidation states of all atoms of a specific element *e* in the test set.

(2) $$P_{E}=\frac{N_{e}^{os}}{N_{e}}$$

where $N_{e}^{os}$ was the number of atoms with element type *e* that have correct predicted oxidation states; *N*_*e*_ was the total number of atoms with element type *e* in all compositions.

Element‐family accuracy *P*_*F*_ measures the percentage of correctly predicted oxidation states of all atoms of a specific elemental family, such as metals in all the compounds of the test set.

(3) $$P_{F}=\frac{N_{e_{i}\in F}^{os}}{N_{e_{i}\in F}}$$

where $N_{e_{i}\in F}^{os}$ was the number of atoms with element type *e*_*i*_ belonging to element family *F* that have correct predicted oxidation states; $N_{e_{i}\in F}$ was the total number of atoms with element type *e*_*i*_ belonging to element family *F* in all compositions.

Compound accuracy *P*_*C*_ measures the percentage of compounds in the test set that have all their atomic oxidation states predicted correctly.

(4) $$P_{C}=\frac{N_{comp}^{os}}{N_{comp}}$$

where $N_{comp}^{os}$ was the number of compounds in the test set with all oxidation states assigned correctly; *N*_*comp*_ was the total number of compounds in the test set.

Compound Average Site Accuracy (CASA) *P*_*CASA*_ measures the average of the percentages of correctly predicted oxidation states for each compound in the test set.

(5) $$P_{CASA}=\frac{\sum_{i}^{N_{comp}}\left(N_{atom\in comp_{i}}^{os}/N_{atom\in comp_{i}}\right)}{N_{comp}}$$

where $N_{atom\in comp_{i}}^{os}$ was the number of atoms with corrected predicted oxidation states for compound *i*. $N_{atom\in comp_{i}}$ was the total number of atoms in compound *i*. *N*_*comp*_ was the total number of compounds in the test set.

The prediction performance could also be evaluated by calculating the percentage of formulas that were charge‐neutrality based on the predicted oxidation state assignments *P*_*CN*_.

(6) $$P_{CN}=\frac{N_{comp}^{CN}}{N_{comp}}$$

where $N_{comp}^{CN}$ was the number of charge‐neutral compounds in the test set with all oxidation states assigned; *N*_*comp*_ was the total number of compounds in the test set.

### 2.5 Statistic Analysis

Despite conducting multiple experiments, one consistently observed low variances of the model's OS prediction performances over different datasets, so only single‐run experimental results were shown here for simplicity.

## 3 Results and Discussion

We first train a BERTOS model using the cleaned OS‐ICSD‐CN training set with 31 827 samples with unique compositions and evaluate its performance on the OS‐ICSD‐CN test set with 3724 unique compositions each labeled with ICSD‐provided oxidation state labels.

### 3.1 Overall Performance

We first check the atomic site level accuracy *P*_*S*_, which measures the percentage of correctly predicted oxidation states of all atoms in all compositions of the test set. Out of 190 468 atomic sites, our algorithm achieves 96.27% accuracy (all elements are counted), which is close to the 98.1% accuracy evaluated for only 994 metal atom oxidation states in 532 ionic and coordination compounds using structure‐based features and Random Forest Classifier.^[^ ^10^ ^]^ Our model even beats the structure‐based ML models for the Mn ion oxidation state prediction^[^ ^9^ ^]^ with the accuracy of 94% and 95% for Gaussian Naive Bayes Classifier and Decision Tree Classifiers tested only on Mn compounds. This result is surprising as our model only uses composition as input information. We then calculate *P*_*C*_, the percentage of test materials that have all their atomic sites with correctly predicted oxidation states. The accuracy *P*_*C*_ reaches 87.76%. In contrast, when we use Pymatgen's oxid_state_guess function, only 4.49% of the test samples can be assigned definite oxidation states. We further calculate the compound level average site accuracy *P*_*CASA*_, which reaches 97.16%.

### 3.2 Element Family Performance

**Figure 2:**
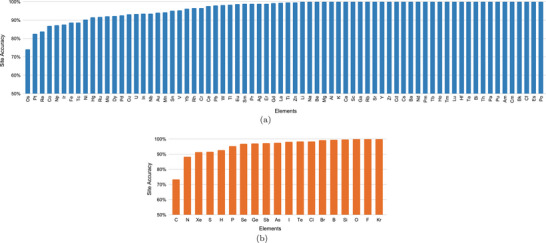
a) Site OS prediction accuracy for metal elements. b) Site OS prediction accuracy for nonmetal elements.

Since different elements take on different sets of possible oxidation states including those common OS ones, it is interesting to check our model performance for different element families. We first calculate the overall *P*_*F*_ accuracy of all metal element sites, which reaches 97.12%, which is slightly lower than 98.1%, the accuracy of the structure‐based machine learning model as reported in Ref. [10]. This unexpected small gap to the structure‐based model performance makes our model to be very useful for screening hypothetical materials compositions designed by deep generative models.^[^ ^3^ ^]^ We then check the performance *P*_*E*_ for individual element OS prediction of metal elements. First, we find that for all elements in the alkali metals and alkaline earth metals except Li (99.93%), K (99.96%), Fr (no data), and Ra (no data), our model achieves 100% site accuracy, reflecting that our model learns to assign oxidation states to these typically ionic elements with high confidence, which is consistent with the chemical understanding of these elements and their assigned OS. Next, we check the elements with low prediction accuracy on the left part of **Figure** **2**. Out of the elements with less than 90% accuracy, a majority of them are transition metals including Os (74.07%), Pt (82.54%), Re (83.90%), Co (86.89%), Np (87.30%), Ir (87.50%), Fe (88.63%), and Tc (88.68%). The relatively low performance for these transition metal elements reflects our model's learned chemistry is consistent with known literature. The other three elements with lower performance are from the Ac family including Fm (no data), Md (no data), No (no data), Lr (no data), and Np (87.30%). We also find in general our model has higher OS prediction performance for the post‐transition metal elements compared to transition metals. These post‐transition metal elements include Sn (95.12%), Pb (98.00%), Ga (100%), Bi (100%), Po (100%), and Al (100%).

In addition, we calculate the overall *P*_*F*_ accuracy for all nonmetal element sites (including the metalloid elements), which reaches 96.05% out of 150 778 sites, and the performance figure is shown in Figure 2. We then check *P*_*E*_ for the nonmetal elements with high performance, and find the OS prediction accuracy of the following elements can hit more than 99%: Br (99.30%), B (99.36%), Si (99.76%), O (99.90%), F (99.95%), and Kr (100%). There are only two non‐mental elements, C (77.38%) and N (88.20%) with site accuracy less than 90%, which can be explained by the fact that C and N have more common oxidation states than most other elements, which makes it difficult for our deep learning model to learn the OS assignment rules. Other than that, the remainder of the nonmetal elements achieves a high site accuracy between 90% and 99%.

### 3.3 Material Family Performance

**Figure 3:**
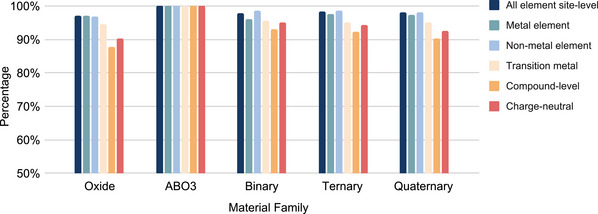
OS prediction performance of BERTOS over different material families. Except for the compound level and charge‐neutrality accuracy (*P*_*C*_ and *P*_*CN*_, respectively), all other performance criteria are at the site level (*P*_*S*_ for all element site level and *P*_*F*_ for the metal element, nonmetal element, and transition metal element).

We further examine how the OS prediction performance on a specific material family varies for specific material families. We calculate the performances of our model for oxides, *ABO*_3_, binary, ternary, and quaternary compounds with the results shown in **Figure** **3**. First, we check the OS performance for the binary, ternary, and quaternary materials. For all element types, BERTOS achieves high accuracy *P*_*S*_ , ranging from 97.77% for binary to 98.03% for quaternary compounds. Comparing the metal and nonmetal elements, the nonmetal elements tend to have higher prediction accuracy *P*_*F*_ across all three families. For metal elements, the performance of ternary and quaternary families is higher than that of the binary compounds. We further find that the OS prediction performance for transition metals is lower than that for the nonmetals and for all metal elements, which is consistent with our chemical knowledge that transition elements are very flexible to form complex compounds. At the compound level, all three families have accuracy *P*_*C*_ greater than 90%. We also check the charge‐neutrality accuracy *P*_*CN*_, which measures what percentage of compounds that are charge‐neutral as annotated in the OS‐ICSD‐CN dataset is still charge‐neutral as predicted by BERTOS. The *P*_*CN*_ of our algorithm for binary, ternary, and quaternary families are 95.02%, 94.39%, and 92.45%, respectively.

We further explore if BERTOS can achieve higher performance for more specialized materials families, so we check the oxide materials and find that BERTOS achieves high performance for all elements, metal elements, nonmetal elements, transition metal, compound level, and charge neutrality, but cannot be higher than binary, ternary, and quaternary compounds. However, when we check the OS prediction performance of BERTOS on the *ABO*_3_ materials, we find that all the performances are 100%, which is much higher than for all other materials families, demonstrating that the OS prediction performance can vary by material families, where some of them are easier to predict as there are stronger OS rules.

**Figure 4:**
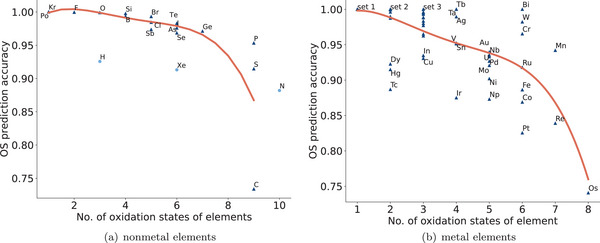
OS prediction performance versus no. of oxidation states. a) nonmetal elements. The elements, H, Xe, and N, are apparent outliers that have lower OS prediction performance compared to those elements with similar numbers of accessible OS. b) metal elements. *set* 1 includes 19 elements with only 1 OS, which are Be, Mg, Al, K, Ca, Rb, Sr, Cd, Cs, Ba, Pm, Ho, Tm, Lu, Hf, Am, Cm, Cf, and Es, and all of them have 100% prediction accuracy. *set* 2 includes 12 elements with 2 OS, which are Eu, Er, Zn, Li, Na, Sc, Ga, Y, Th, Pa, Pu, and Bk in descending order of the prediction accuracy. *set* 3 includes 12 elements with 3 OS, which are Yb, Rh, Ce, Pb, Tl, Sm, Pr, Gd, La, Ti, Zr, and Nd in descending order of the prediction accuracy. For metal elements, while there is a pattern that the more the number of accessible oxidation states an element has, the more difficult it is to predict its oxidation states, there are many exceptions. The elements Tc, Ir, Np, and Pt are apparent outliers that have lower OS prediction performance compared to those elements with similar numbers of accessible OS. Bi, W, Cr, and Mn instead have higher OS prediction accuracy compared to others with a similar number of accessible OS.

We then check how the number of oxidation states of elements affects their OS prediction performance. Our assumption is that the smaller the number of oxidation states an element may have, the easier it is to predict its OS. **Figure** **4**a shows how the nonmetal element OS prediction performance of our model varies as regard to the number of possible oxidation states for a given element. We find that other than a few exceptions, there is a clear trend that when the element's OS set grows larger, it becomes more difficult to predict its OS. For the exception set of elements (i.e., C, S, P, and N) with more than 9 possible OS according to the ICSD annotations, the prediction accuracies don't follow this trend but range from 73.38% to 95.33%. Investigating the reason, the accuracy for elements with much more OS is influenced by their different degrees of chemical activities rather than simply by the number of OS. While there is still a trend that it is more difficult to predict their OS for elements with more OS, the variation is much higher: even for elements with only 5 OS such as Np, our model only achieves an accuracy of 87.30%, but the Mo element with the same number of OS (5 possible OS) can achieve 92.07%.

**Figure 5:**
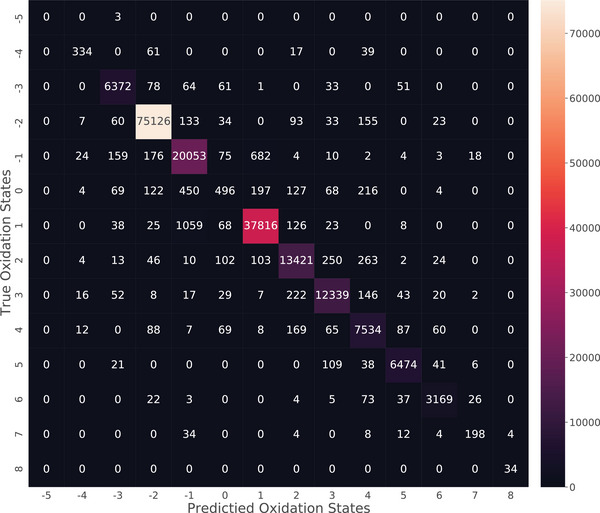
Confusion matrix of OS prediction evaluated on the OS‐ICSD‐CN test set. The x‐axis represents predicted oxidation states, and the y‐axis represents true oxidation states. The color scale on the right shows the relationship between the number of samples distribution and the shade of the color, which is the lighter the color, the more samples there are. Then, the numbers in the diagonal mean the number of the correct predictions.

To explore the performance of OS predictions on each oxidation state (from ‐5 to +8), we plot the confusion matrix of the OS predictions for the OS‐ICSD‐CN test set (**Figure** **5**), and the overall accuracy of the predictions can hit 96.27%. According to the shade of color, the oxidation states of most atomic sites concentrate between ‐3 and +6, and only a few atoms have OS distributed among ‐5, ‐4, 0, +7, and +8. Of all the OS, ‐2 is found in a total of 75,126 sites, which is the largest one with BERTOS prediction accuracy as high as 99.29%, the highest performance among all the OS. One of the possible reasons is that there are a lot of oxides and sulfides in our datasets, in which the oxygen and sulfur elements usually exit in their common oxidation state of ‐2. We then check the OS prediction performance of BERTOS over other OS, and find with the exception of ‐5 (3 samples, 0%), ‐4 (451 samples, 74.06%), 0 (1753 samples, 28.29%), and +7 (264 samples, 75%), the prediction accuracy of all other OS is more than 94%, which is reasonable since all of the OS with poor accuracy including ‐4, 0, and +7 are the ones with the fewest existing samples. In addition to showing the overall OS prediction performance with regard to the atom sites, we also divide the atomic sites of the test set into metal and nonmetal ones and show their confusion matrices (see Figures S1 and S2, Supporting Information). The overall accuracy of the metal atomic site OS reaches 97.12%, and the nonmetal one reaches 96.05%. The most accurate OS for metal sites are +1, +2, and +3 while the most accurate OS for nonmetal sites are ‐2, ‐1, +1, and +4.

### 3.4 Case Studies of OS Prediction

To illustrate the power of our BERTOS model for predicting non‐trivial OS, we conduct a set of case studies for three categories of compounds. **Table** **1** (first two columns) shows the compounds with accurately predicted OS that contain at least one transition metal element combined with one non‐transition metal and with a total number of elements ranging from 3 (ternary) to 5(quinary). The OS column shows the accurately predicted oxidation states with the corresponding elements in the composition on the left column. The bold numbers show that the ground truth OS of the elements contains multiple common OS, which makes it non‐trivial to estimate by human experts. First, for *Nb*_4_ *Bi*_4_ *O*_16_, all OS are the only common OS of the composition elements, which both BERTOS and chemists can estimate correctly with ease. A more complex situation is shown in *Rb*_4_ *Cd*_4_ *Cl*_12_ and *Sr*_4_ *La*_4_ *I*_16_, in which the *Cl* and *I* elements have multiple common OS. Assuming these two elements each have two common OS, there are four possible combinations of common OS, for which our algorithm predicts correctly which is four times better compared to random guess. This performance is even higher when considering that the elements may also take non‐common OS. For quarternary compounds, our model predicts the OS correctly for four example compounds with two elements each taking ⩾2 common OS. Particularly, we find that our BERTOS predicts the OS (1, 1, ‐1, ‐1) of *Rb*_4_ *Cu*_15_ *I*_7_ *Cl*_12_ correctly, which contains three elements (*Cu*, *I*, *Cl*) all with multiple common OS. For quinary materials, our model also accurately predicts the OS of those that contain one or two elements with multiple common OS. Overall, for this whole category, our model achieves an average compound site accuracy *P*_*CASA*_ of 98.3% and a compound accuracy *P*_*C*_ of 91.16%, which indicates the percentage of compounds in the test set that have all their atomic oxidation states predicted correctly.

**Table 1:** Case studies of complex OS predictions

	1 transition metal + 1 non‐transition metal		2 transition metals	
	Composition	OS	Composition	OS
	*Nb*_4_ *Bi*_4_ *O*_16_	5,3,‐2	*Hg*_4_ *W*_4_ *O*_16_	**2**,**6**,‐2
	*Rb*_4_ *Cd*_4_ *Cl*_12_	1,2,**‐1**	*La*_6_ *Cr*_6_ *O*_18_	3,**3**,‐2
Ternary	*Dy*_1_ *Cu*_1_ *Te*_2_	3,**1**,**‐2**	*Nb*_8_ *Ni*_16_ *O*_36_	5,2,‐2
	*Sr*_4_ *La*_4_ *I*_16_	2,2,‐1	*La*_4_ *W*_4_ *O*_18_	3,**6**,‐2
	*Na*_8_ *Pt*_4_ *Se*_8_	1,**2**,**‐2**	*Zr*_4_ *Mo*_8_ *O*_32_	4,**6**,‐2
	*Sr*_5_ *Mn*_4_ *CO*_13_	2,**3**,**4**,‐2	*Li*_2_ *La*_4_ *Re*_2_ *O*_12_	1,3,**5**,‐2
	*Rb*_8_ *Ti*_8_ *As*_8_ *O*_40_	1,**4**,**5**,‐2	*Ti*_4_ *Tl*_8_ *W*_12_ *O*_48_	**4**,**1**,**6**,‐2
Quatenary	*Cs*_4_ *Ag*_2_ *Sb*_2_ *S*_8_	1,1,**5**,**‐2**	*Sr*_4_ *Fe*_2_ *Mo*_2_ *O*_12_	2,**3**,**5**,‐2
	*Rb*_4_ *Cu*_15_ *I*_7_ *Cl*_12_	1,**1**,**‐1**,**‐1**	*Cd*_4_ *Mo*_4_ *P*_4_ *O*_24_	2,**5**,**5**,‐2
	*Cd*_4_ *Bi*_4_ *S*_8_ *Cl*_4_	2,3,**‐2**,**‐1**	*LaFe*_4_ *Bi*_3_ *O*_12_	3,**3**,3,‐2
	*Na*_10_ *Sc*_2_ *H*_8_ *C*_8_ *O*_28_	1,3,1,**4**,‐2	*BaNdMnCoO*_5_	2,3,**3**,**2**,‐2
	*K*_8_ *Cr*_4_ *H*_8_ *Cl*_20_ *O*_4_	1,**3**,1,**‐1**,‐2	*Ba*_2_ *NdNbCu*_2_ *O*_8_	2,3,5,**2**,‐2
Quinary	*Na*_2_ *Cu*_4_ *H*_6_ *S*_4_ *O*_20_	1,**2**,1,**6**,‐2	*Na*_3_ *DyTi*_2_ *Nb*_2_ *O*_12_	1,3,**4**,5,‐2
	*Na*_8_ *H*_5_6*Pt*_4_ *C*_16_ *O*_64_	1,1,**4**,**3**,‐2	*BaMg*_2_ *V*_6_ *Cu*_8_ *O*_26_	2,2,**5**,**2**,‐2
	*MgHg*_3_ *H*_12_ *Cl*_8_ *O*_6_	2,**2**,1,**‐1**,‐2	*Na*_2_ *Ni*_4_ *Mo*_4_ *H*_6_ *O*_20_	1,2,**6**,1,‐2

Materials with two transition metal elements are even more difficult to predict their OS. Table 1 (3rd and 4th columns) shows the examples with accurately predicted OS that contain at least two transition metal elements. Similar to the cases discussed above, BERTOS accurately predicts the OS for this family of materials with 1/2/3 elements each with multiple common OS. In addition, we find that for *Sr*_4_ *Fe*_2_ *Mo*_2_ *O*_12_, and *Cd*_4_ *Mo*_4_ *P*_4_ *O*_24_, our model accurately predicts the OS of *Mo* to be 5, which is NOT its common OS. Another challenge is when a compound has multiple transition metal elements each with multiple common OS and there are multiple combinations of the common OS that satisfy the charge‐neutrality criterion, it is difficult for a chemist to guess its true OS. This case happens with *BaNdMnCoO*_5_, which has the ground truth OS of 2,3,3,2,‐2. Here, *Mn* and *Co* can either take (3, 2) or (2, 3), both leading to a charge‐neutral state for the compound, but our model accurately predicts the ground truth OS as (3, 2) for *Mn* and *Co*. For this family of materials, our BERTOS model achieves an average compound site accuracy *P*_*CASA*_ of 98.02% and a compound accuracy *P*_*C*_ of 89.26%. We also estimate the OS prediction performance for materials with at least one transition metal and one nonmetal elements with a total of 1753 test samples. Our model achieves an average compound site accuracy *P*_*CASA*_ of 96.29% and a compound accuracy *P*_*C*_ of 84.54%. The case study examples are shown in Table S4 (Supporting Information).

### 3.5 BERTOS for Screening Hypothetical Materials Compositions

**Figure 6:**
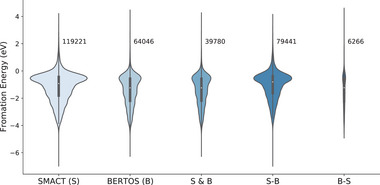
Comparison of the formation energy distribution of binary and ternary candidate materials filtered by our BERTOS model trained on the OS‐ICSD‐CN dataset and by the enumerative charge‐neutrality check algorithm of SMACT. For SMACT (S), the compositions are filtered by the SMACT enumeration algorithm. For BERTOS (B), the compositions are selected by BERTOS. For S&B, the compositions are the common part in S and B. For *S* − *B*, the compositions are from S, but not in B. For *B* − *S*, the compositions are from B, but not in S.

Previously, researchers only use Pymatgen's oxid_state_guess or the enumerative algorithm of the SMACT package^[^ ^5^ ^]^ for screening charge‐neutral compositions generated by the deep generative models.^[^ ^6^ ^]^ However, one drawback of these oxidation state check methods is that they only consider mathematically possible combinations of accessible oxidation states of the constituent elements and neglect their preferred oxidation states in a given composition. We use our BERTOS model for large‐scale hypothetical material screening. We first use the BLMM generative model^[^ ^6^ ^]^ to generate one million hypothetical compositions. After duplicate removal compared to the OQMD,^[^ ^26^ ^]^ ICSD, and Materials Project^[^ ^27^ ^]^ databases, there are 635 064 new compositions left. We then filter out the binary and ternary compositions with the no. of atoms less than or equal to 30, leading to 175 798 compositions. Next, we apply two approaches to screen out charge‐neutral compositions. First, as a baseline, we use the exhaustive enumeration algorithm for charge‐neutrality checking as implemented in the SMACT package to filter out 119 221 compounds. Similarly, we apply our BERTOS to predict the oxidation states of the 175 798 compositions, select 64 046 hypothetical candidates, and compute the intersections and differences with those filtered by SMACT. We then predict the formation energies using the Roost model^[^ ^25^ ^]^ (See Experimental Section for details) for the compositions screened by our BERTOS and by SMACT, and plot their distributions as shown in the violin plots (A hybrid of a box plot and kernel density plot for visualizing data distribution^[^ ^28^ ^]^) of **Figure** **6**. First, we find that compositions selected by SMACT (*S*) are located more at the higher energy region compared to those selected by BERTOS (*B*). There are 39,780 shared compositions (S&B) between these two models, and the shape of S&B in Figure 6 is very similar to the shape of *B*, which tends to have lower formation energies. We also show the distributions of those compositions that only appear in the SMACT set but not in the BERTOS set (S‐B) and compare it to those that only appear in the BERTOS set but not in the SMACT set (B‐S). We find that SMACT tends to get more samples located at the higher end of the energy band, while the 6266 unique samples selected by BERTOS tend to be located evenly toward the lower‐energy area. These results show that BERTOS provides more stringent criteria and can select higher‐quality hypothetical material candidates. With high‐quality hypothetical compositions, one can use the modern template‐based crystal structure prediction algorithms to obtain their structures^[^ ^29^, ^30^ ^]^ and use DFT calculations for further validation.

### 3.6 Discussion

Given a material's structure, the chemical theories based bond valence sum method, structure descriptors based machine learning methods, and even DFT calculations may be used to obtain the actual oxidation states or charges of atomic sites. However, in computational materials discovery of novel materials, the structure is not even known preventing us from using any of these existing methods. Here, we propose the first only composition‐based oxidation state prediction model for all element types based on the deep learning transformer language model neural networks, which is built for dealing with token sequence information with variable lengths. We formulate this oxidation state prediction problem as the token classification problem in natural language processing and surprisingly find that despite the fact that our encoding of the compositions does not take any atomic properties into account except for the elemental symbols themselves encoded with one‐hot vectors, our transformer neural networks achieve superb performance for oxidation state prediction. In our analysis of 635 051 hypothetical compositions generated by the CrystalTransformer neural network generator model,^[^ ^6^ ^]^ only 176 555 are predicted to have charge neutrality. However, the Roost model predicts that 602 644 of them have formation energy less than 0. So essentially, our model can save 70.7% of computation time for downstream crystal structure prediction and DFT validation by focusing only on chemically valid hypothetical compositions.

We find that BERTOS' OS prediction performance variations and difficulties are strongly consistent with known chemical intuition: predicting OS for atoms that can adopt multiple oxidation states is more difficult, which is especially true for nonmetal elements. We also find metal atoms are much more difficult to predict their OS compared to the nonmetal ones given the same number of possible oxidation states. We also explore what are common mistakes in predicting the OS of a given element. Using the confusion matrix of BERTOS for carbon element OS prediction as shown in Figure S3 (Supporting Information), we find that two oxidation states, +2 and +4, are the most popular for carbon element in our dataset. For the true OS of +2, there are 2059 samples classified correctly, 190 samples misclassified as +4, and a small number of samples misclassified as ‐4, ‐2, 0, 1, 3. For the true OS of +4, there are 1385 samples classified correctly, 130 samples misclassified as +2, and a small number of samples misclassified as ‐4, ‐2, 0, 3.

One of the important factors that affect the OS prediction performance is the amount of the training set and the distributions of the training and test sets. To investigate this issue, we train four different BERTOS models using the four training sets (See Table S1, Supporting Information) from OS‐ICSD, OS‐ICSD‐CN, OS‐ICSD‐oxide, and OS‐ICSD‐CN‐oxide datasets and test their performance on four different test sets (See Supporting Information for detailed data processing steps to ensure there are no overlaps between the training sets and the test sets). **Table** **2** shows the OS prediction performance of different models over different test sets. First, for the OS‐ICSD test set, the model trained with OS‐ICSD training set achieves the best performance of 96.82%. Since this test set has the most diverse compositions that include those that are not assigned with non‐charge‐neutral oxidation states, it requires the training set also to contain diverse samples. For the OS‐ICSD‐CN test set which contains only samples with charge‐neutral oxidation state assignments by ICSD, the model trained with OS‐ICSD achieves the best performance of 96.28%, and the model trained with OS‐ICSD‐CN has a similar accuracy of 96.27%. For the OS‐ICSD‐oxide test set containing only oxide samples, the OS‐ICSD‐oxide trained model achieves the best performance of 97.61% as expected since the OS‐ICSD‐oxide training set and test set have more similar data distributions. Finally, for the charge‐neutral oxide test samples in the OS‐ICSD‐CN‐oxide test set, the model trained with the OS‐ICSD‐oxide dataset achieves the best performance of 97.14%, slightly beats the performance of 96.97% of the model trained only with charge‐neutral oxides in the OS‐ICSD‐CN‐oxide training set with the similar argument for OS‐ICSD‐oxide test set. The reason may be that the OS‐ICSD‐oxide contains much more training samples (30 519) in addition to those oxides (20 601) in the OS‐ICSD‐oxide. Those non‐CN training samples can be used to prevent the overfitting of the model, which can lead to better performance. Overall, we find that the OS prediction performance of BERTOS for a given test set is the best or close to the best when the model is trained with a training set with similar compositions, indicating that more specialized training sets can produce a higher performance for specialized test sets. However, increasing the training sets with additional diverse samples may also increase the OS prediction performance, especially when the training sets are relatively small. We also compare the performance of the GPT2‐based OS prediction model with BERTOS, both of which are trained on the OS‐ICSD‐CN dataset. And we find that the atomic site accuracies of GPT2‐based model are 91.15%, 90.14%, 94.21%, and 93.23% on the test sets of OS‐ICSD, OS‐ICSD‐CN, OS‐ICSD‐oxide, and OS‐ICSD‐CN‐oxide, respectively. Compared to the accuracies of BERTOS (See 2nd row in Table 2), the GPT2‐based model has worse performance.

**Table 2:** OS prediction performance versus datasets

Train\Test	OS‐ICSD	OS‐ICSD‐CN	OS‐ICSD‐oxide	OS‐ICSD‐CN‐oxide
OS‐ICSD	**96.82%**	**96.28%**	97.51%	97.11%
OS‐ICSD‐CN	95.92%	**96.27%**	96.60%	96.95%
OS‐ICSD‐oxide	95.78%	94.96%	**97.61%**	**97.14%**
OS‐ICSD‐CN‐oxide	94.95%	94.85%	96.70%	96.97%

We also evaluate our BERTOS model's capability to handle the OS assignment problem of complex systems. For example, an oxide (e.g., *NiFeO*_*X*_) can have mixed oxidation states with respect to the local environments with different x. In this case, *NiFeO*_*X*_ is not a specific compound with a well‐defined chemical formula, so it is difficult to provide a definitive answer regarding its oxidation states. Ni, Fe, and O are all elements that commonly have multiple oxidation states. Ni (nickel) can have oxidation states of +2 or +3, with +2 being the most common. Fe (iron) can have oxidation states of +2 or +3, with +2 being more common in compounds, and +3 more common in aqueous solutions. O (oxygen) typically has an oxidation state of ‐2 in compounds. Without more information about the specific compound or system being referred to, it is difficult to determine the oxidation states of Ni, Fe, and O in *NiFeO*_*X*_. However, we found that our algorithm can differentiate these two cases, correctly predicting the OS for Ni and Fe to be +2 for the case of *NiFeO*_2_ while the OS for Ni and Fe to be +3 for the case of *NiFeO*_3_, indicating that our model has certain capability to differentiate mixed oxidation states if the local environment variation is reflected in their compositions. For the case of polymorphs of a given composition, our model tends to predict the most common oxidation configurations. Without additional structure information, it is impossible for our composition based model to differentiate OS differences due to the different structural local environments. One possible solution is to first predict their structures and then apply structure‐based OS prediction. Currently, our model only outputs the OS states with maximum probability scores for each atomic site. It is possible for our model to report multiple suboptimal OS assignments that may be used to address the cases with multi‐OS for a single element in a crystal. On the other hand, our analysis of ICSD dataset showed that out of the 132 972 ICSD materials we studied, only 9129 (6.8%) materials contain elements with two or more OS states.

There are several ways our knowledge‐agnostic deep learning model may be improved. For example, it may be useful to include the valence electrons and orbits information of the elements and their common oxidation states into the element encoding. We may also conduct post‐processing to fix the model prediction errors using known chemical rules such as charge‐neutrality. We recognize that the actual oxidation states of atoms are also strongly dependent on their chemical environment so the structure information is also important. However, it is surprising that our BERTOS achieves such high accuracy without the structure information. In addition, minority elements have worse performance than majority elements because there is an imbalanced element distribution in our datasets. This is a challenging issue, but we find that we cannot use standard synthetic samples and resampling strategy in OS prediction. While generating synthetic samples is commonly used to address the imbalance issue, we cannot force data balanced in this way: the oxidation state of an atom in a compound has its own meaning due to electrons gained or lost, which means that we cannot randomly generate synthetic samples as it breaks the chemical validity. Additionally, the resampling process can only be conducted over formulas, which cannot just increase the percentages of minority elements without increasing the percentages of majority elements in the dataset. So the strategy of resampling cannot achieve the goal of balancing the data distribution. Further investigation is needed to address the data imbalance issue that may improve our model performance.

One unique advantage of our transformer‐based OS prediction model is that all its predictions come with uncertainty quantification (the probability scores) without using those tedious ensemble‐model‐based uncertainty estimation methods.^[^ ^31^ ^]^ These confidence scores of probability assigned to the predicted OS of each atomic site make it easy for users to interpret the prediction results. As a demonstration of its usage, check the probability distributions of those correctly predicted OS and those incorrectly predicted OS and find that the average probability scores are much lower for the incorrect OS predictions compared to the correct OS predictions.

## 4 Conclusion

We proposed a transformer language model (BERT) based deep neural network, BERTOS, for composition‐based oxidation state prediction of inorganic materials. Extensive experiments have shown the unexpectedly high performance of our BERTOS models for assigning oxidation states to both metal and nonmetal elements given only the composition/formula of a material. The model's performances over different families of elements and materials are all consistent with known chemistry knowledge. The high correlation between the number of accessible oxidation states of nonmetals and the oxidation prediction performance of our models is interesting, as we did not observe such a strong correlation for metal elements. Since our model did not take into account any atomic attributes such as the valence electrons and orbital information, the self‐emergence of the chemical knowledge learned by the knowledge‐agnostic deep learning language models illustrates their great potential to explore new chemical knowledge. We still wonder and expect that when the elemental valence electron information and the orbital attributes are introduced to our model, the performance may be further improved or at least improved for a small training dataset. Compared to previous structure‐based oxidation state prediction models, our composition‐based oxidation state prediction models will have a large potential for screening millions of hypothetical new material compositions from generative design.^[^ ^6^, ^7^ ^]^ The web app for BERTOS can be accessed at http://www.materialsatlas.org/bertos.

## Conflict of Interest

The authors declare no conflict of interest.

## Author Contributions

J.Hu performed conceptualization. N.F., J.H., Y.F., G.M., and J.Hu performed methodology. N.F., J.H., Y.F., and J.Hu acquired software. J.Hu acquired resources. J.H., N.F., Y.F., J.Hu, and H.Z. prepared the original draft. J.Hu and N.F. wrote—reviewed and edited. N.F., J.H., and J.Hu performed visualization, supervision, and performed funding acquisition.

## Supporting information

Supporting Information

## Data Availability

The pretrained models and datasets are freely available at http://www.github.com/usccolumbia/BERTOS.
