# Supplementary material for: Composition Based Oxidation State Prediction of Materials Using Deep Learning Language Models
Source: Adv Sci (Weinh). 2023 Aug 7;10(28):2301011. doi: 10.1002/advs.202301011 (PMC10558692; doi:10.1002/advs.202301011)
Supplement: Supplementary file 1 — Supporting Information [file ADVS-10-2301011-s001.pdf]

ADVANCED SCIENCE Open Access

Supporting Information

for *Adv. Sci.*, DOI 10.1002/advs.202301011

Composition Based Oxidation State Prediction of Materials Using Deep Learning Language Models

*Nihang Fu, Jeffrey Hu, Ying Feng, Gregory Morrison, Hans-Conrad zur Loye and Jianjun Hu**

Supplementary file for *Composition based oxidation state prediction of materials using deep learning language models*

Jeffrey Hu, Nihang Fu, Ying Feng, Gregory Morrison, Hans-Conrad zur Loye, Jianjun Hu

July 10, 2023

1 Dataset preparation

Our training and test datasets are prepared using the following process. We obtain 151,707 ICSD CIF structure files, and each CIF is annotated with oxidation states. However, there are many entries such as intermetallic materials are assigned 0 oxidation states (OS) to all their atoms. We thus first exclude all structures with 0 oxidation state assignment. We also exclude those materials with fractional oxidation states or those materials with >200 atoms. Since ICSD contains many CIF structure files that neglect the hydrogen atoms, we develop an algorithm to add the hydrogen atoms back to the structures along with their OS. Considering that one composition may correspond to multiple structural phases, we select for the formula the oxidation state assignment that appear most frequently among all the polymorphism phases. We also exclude those materials only with a single element. Finally, we obtain 52,147 formulas with valid oxidation states to which we name it as the OS-ICSD dataset. We then split this dataset into the training set, the validation set, and the test set using the 85%:5%:15% ratio.

To examine whether specialized material families can be used to train a more accurate OS prediction model, we first exclude those formulas with fractional atomic numbers and then select only charge-neutral (CN) formulas from the OS-ICSD's training, validation, and test sets, which generate our OS-ICSD-CN dataset. We further select only oxide materials from the OS-ICSD dataset from its training, validation and test sets to compose our OS-ICSD-oxide dataset. Finally, we compose our OS-ICSD-CN-oxide dataset by selecting charge-neural oxides from OS-ICSD's training, validation, and test sets. Our data construction procedure ensures that all four test sets never overlap with all training and validation sets so that the models trained with four different training sets can be tested using any of the four test sets. The details of the final four datasets are shown in Table S1.

Table S1: Statistics of training and test datasets

Dataset	Training	Validation	Test
OS-ICSD	44324	2608	5215
OS-ICSD-CN	31827	1873	3724
OS-ICSD-oxide	30519	1764	3603
OS-ICSD-CN-oxide	20601	1208	2420

2 OS Distribution in Datasets

We list the distributions of oxidation states of each element in our four datasets.

Table S2: Oxidation state distributions in OS-OCSD, OS-ICSD-CN, OS-ICSD-oxide, and OS-ICSD-CN-oxide datasets

	**OS-ICSD**	**OS-ICSD-CN**	**OS-ICSD-oxide**	**OS-ICSD-CN-oxide**
**H**	1,-1,0	1,-1,0	1,-1,0	1,-1,0
**He**	0	0	0	0

**Li**	1,0	1,0	1,0	1,0
**Be**	2	2	2	2
**B**	-3,-2,0,2,-1,3,1	-3,-2,0,2,-1,3,1	-3,0,2,-1,3,1	-3,0,2,-1,3,1
**C**	-3,0,4,-2,2,-1,3,1,-4	-3,0,4,-2,2,-1,3,1,-4	-3,0,4,-2,2,-1,3,1,-4	-3,0,4,-2,2,-1,3,1,-4
**N**	-3,5,0,-2,4,2,-1,3,1,-5,-4	-3,5,-2,0,4,2,-1,3,1,-5,-4	-3,5,0,-2,4,2,-1,3,1	-3,5,-2,0,4,2,-1,3,1
**O**	-2,-1,0	-2,-1,0	-2,-1,0	-2,-1,0
**F**	-1,0	-1,0	-1,0	-1,0
**Na**	1,-1,0	1,0	1,0	1,0
**Mg**	0,2	0,2	2	2
**Al**	0,1,2,3	0,1,2,3	3	3
**Si**	-3,0,4,-2,2,-1,3,1,-4	-3,0,4,-2,2,-1,3,1,-4	0,4,2,-1,3,1,-4	0,4,-1,3,1,-4
**P**	6,5,-3,-2,4,0,2,-1,3,1,-4	6,5,-3,0,4,-2,2,-1,3,1,-4	6,5,-3,-2,4,0,-1,3,1,-4	6,5,-3,0,4,-2,-1,3,1,-4
**S**	6,7,5,0,-2,4,2,-1,3,1	6,7,5,0,-2,4,2,-1,3,1	6,7,5,-2,0,4,2,-1,3,1	6,7,5,-2,0,4,2,-1,3,1
**Cl**	5,0,4,-1,3,7,1,-4	5,0,4,-1,3,7,1,-4	5,0,4,-1,3,7,1	5,0,4,-1,3,7,1
**Ar**	0	0	0	0
**K**	1,0	1,0	1,0	1,0
**Ca**	0,-1,2	0,2	0,2	0,2
**Sc**	1,2,3	1,2,3	2,3	2,3
**Ti**	1,4,2,3	1,4,2,3	4,2,3	4,2,3
**V**	7,5,0,4,2,3,1	5,0,4,2,3,1	5,0,4,2,3,1	5,0,4,2,3,1
**Cr**	6,5,0,4,2,3,1	6,5,0,4,2,3,1	6,5,0,4,2,3	6,5,0,4,2,3
**Mn**	6,5,0,4,2,-1,3,7,1	6,5,0,4,2,-1,3,7,1	6,5,0,4,2,-1,3,7,1	6,5,0,4,2,-1,3,7,1
**Fe**	6,5,0,4,2,-1,3,1	6,5,0,4,2,-1,3,1	6,5,0,4,2,-1,3,1	6,5,0,4,2,-1,3,1
**Co**	0,4,2,-1,3,1	0,4,2,-1,3,1	0,4,2,-1,3,1	0,4,2,-1,3,1
**Ni**	0,4,2,3,1	0,4,2,3,1	0,4,2,3,1	0,4,2,3,1
**Cu**	0,4,2,3,1	0,4,2,3,1	0,1,2,3	0,1,2,3
**Zn**	0,4,2,3	0,4,2,3	4,2	4,2
**Ga**	0,4,2,3,1	0,4,2,3,1	1,4,2,3	1,2,3
**Ge**	-3,-2,4,0,2,-1,3,1,-4	-3,0,4,-2,2,-1,3,1,-4	0,4,2,-1,-4	0,4,2,-1,-4
**As**	5,-3,-2,0,4,2,-1,3,1	5,-3,-2,4,0,2,-1,3,1	5,-3,0,4,-2,2,-1,3,1	5,-3,0,4,-2,2,-1,3,1
**Se**	6,5,-2,4,0,2,-1,3,1	6,5,-2,4,0,2,-1,3,1	6,5,-2,4,0,2,-1,3,1	6,5,-2,4,0,2,-1,3,1
**Br**	5,0,-1,3,7,1	5,0,-1,3,7,1	5,0,-1,3,7,1	5,0,-1,3,7,1
**Kr**	0,2	0,2	0,2	0,2
**Rb**	1,-1,0	1,-1,0	1	1
**Sr**	2	2	2	2
**Y**	1,4,2,3	1,4,2,3	3	3
**Zr**	1,4,2,3	1,4,2,3	1,4,2	1,4,2
**Nb**	6,5,4,2,3,7,1	7,5,4,2,3,1	6,5,4,2,3	3,4,2,5
**Mo**	6,7,5,0,4,2,3,1	6,7,5,0,4,2,3,1	6,7,5,0,4,2,3,1	6,5,0,4,2,3,7,1
**Tc**	6,5,0,4,2,3,7,1	6,5,0,4,2,3,7,1	6,5,0,4,2,3,7,1	6,5,0,4,2,3,7,1
**Ru**	6,5,8,0,4,2,3,7,1	6,5,8,0,4,2,3,7,1	6,5,8,0,4,2,3,7,1	6,5,8,0,4,2,3,7,1
**Rh**	5,0,4,2,-1,3,1	5,0,4,2,-1,3,1	0,4,2,3,1	0,4,2,3,1
**Pd**	0,4,2,3,1	0,4,2,3,1	0,4,2,3,1	1,4,2,3
**Ag**	0,1,2,3	0,1,2,3	0,1,2,3	0,1,2,3
**Cd**	0,1,2	1,2	0,1,2	2
**In**	0,2,-1,3,1	0,2,-1,3,1	0,2,-1,3,1	0,2,-1,3,1
**Sn**	0,4,2,-1,3,-4	0,4,2,-1,3,-4	0,4,2,-1,3,-4	0,4,2,-1,3,-4
**Sb**	5,-3,-2,4,0,2,-1,3	5,-3,-2,4,0,2,-1,3	5,-3,-2,4,0,-1,3	5,-3,-2,4,0,-1,3
**Te**	6,5,-3,-2,4,0,2,-1,1	6,-3,-2,4,0,2,-1,1	6,-2,4,0,2,-1,1	6,-2,4,0,2,-1,1
**I**	7,5,0,-1,3,1	7,5,0,-1,3,1	5,0,-1,3,7,1	5,0,-1,3,7,1
**Xe**	6,8,0,4,2,3	6,8,0,4,2,3	6,8,0,2,3	6,8,0,2,3
**Cs**	1,0	1,0	1,0	1,0
**Ba**	0,2	2	0,2	2
**La**	0,4,2,3,1	0,4,2,3,1	2,3	2,3

**Ce**	0,4,2,-1,3	4,2,3	4,2,3	4,2,3
**Pr**	4,2,3	4,2,3	4,2,3	4,2,3
**Nd**	0,4,2,3	0,4,2,3	0,4,2,3	0,4,2,3
**Pm**	3	3	3	3
**Sm**	1,4,2,3	1,4,2,3	1,4,2,3	1,4,2,3
**Eu**	0,4,2,3,1	0,4,2,3	0,4,2,3,1	0,4,2,3
**Gd**	0,4,2,3,1	0,4,2,3,1	4,2,3	4,2,3
**Tb**	0,4,2,3,1	0,4,2,3,1	0,4,2,3	0,4,2,3
**Dy**	4,2,3	4,2,3	2,3	2,3
**Ho**	4,2,3	4,2,3	2,3	2,3
**Er**	0,4,2,3	0,4,2,3	0,2,3	0,2,3
**Tm**	4,2,3	4,2,3	3	3
**Yb**	0,4,2,3	0,4,2,3	2,3	2,3
**Lu**	4,2,3	4,2,3	3	3
**Hf**	4,2,3	4,2,3	4	4
**Ta**	6,5,4,2,3,1	6,5,4,2,3,1	6,5,4,2,1	6,5,4,2,1
**W**	6,5,0,4,2,3,1	6,5,0,4,2,3	6,5,0,4,2,3	6,5,0,4,2,3
**Re**	6,5,0,4,2,3,7,1	6,5,0,4,2,3,7,1	6,5,0,4,2,3,7,1	6,5,0,4,2,3,7,1
**Os**	6,7,5,8,0,4,2,3,1	6,7,5,8,0,4,2,3,1	6,7,5,8,0,4,2,3,1	6,7,5,8,0,4,2,3,1
**Ir**	6,5,0,4,-2,2,3,1	6,5,0,4,2,3,1	6,5,0,4,3,1	6,5,0,4,3,1
**Pt**	6,5,0,4,-2,2,3,1	6,5,0,4,-2,2,3,1	6,5,0,4,-2,2,3	6,5,0,4,-2,2,3
**Au**	5,2,-1,3,1	5,2,-1,3,1	1,-1,2,3	1,-1,2,3
**Hg**	1,2,0	1,2,0	1,2,0	1,2,0
**Tl**	0,2,-1,3,1	0,2,-1,3,1	0,1,-1,3	0,1,-1,3
**Pb**	0,4,2,-1,3,1,-4	0,4,2,-1,1,-4	0,4,2,3,1,-4	0,4,2,1,-4
**Bi**	5,-3,0,4,-2,2,-1,3,1	5,-3,0,4,-2,2,-1,3,1	5,-3,0,4,-2,2,3	5,-3,0,4,-2,2,3
**Po**	4,2	4,2	4	4
**Ra**	2	2	null	null
**Ac**	3	3	3	3
**Th**	6,5,4,2,3	6,5,4,2,3	4,2	4
**Pa**	3,4,5	3,4,5	4,5	4,5
**U**	6,5,4,2,3,1	6,5,4,2,3,1	3,4,6,5	3,4,6,5
**Np**	6,5,4,2,3,7	6,5,4,2,3,7	6,5,4,2,3,7	6,5,4,2,3,7
**Pu**	6,5,4,2,3,7	6,5,4,2,3,7	6,5,4,2,3,7	6,5,4,2,3,7
**Am**	4,2,3,5	4,2,3,5	4,2,3,5	4,2,3,5
**Cm**	4,2,3	4,2,3	4,2,3	4,2,3
**Bk**	4,2,3	4,2,3	4,2,3	4,2,3
**Cf**	4,2,3	4,2,3	4,3	4,3
**Es**	3	3	3	3

3 BERTOS Network structure and hyper-parameter

We list the network hyperparameters and the training parameters of our BERTOS network.

Table S3: Hyperparameters of BERTOS network

**Network Parameters**		**Training Parameters**	
Vocabulary Size	123	Batch Size	256
Hidden Size	120	Epochs	500
Max Position Embeddings	200	Learning Rate	1e-3
Attention Heads	4		
Hidden Layer	12		

4 Figures

We plot confusion matrices for non-metal and metal element OS predictions.

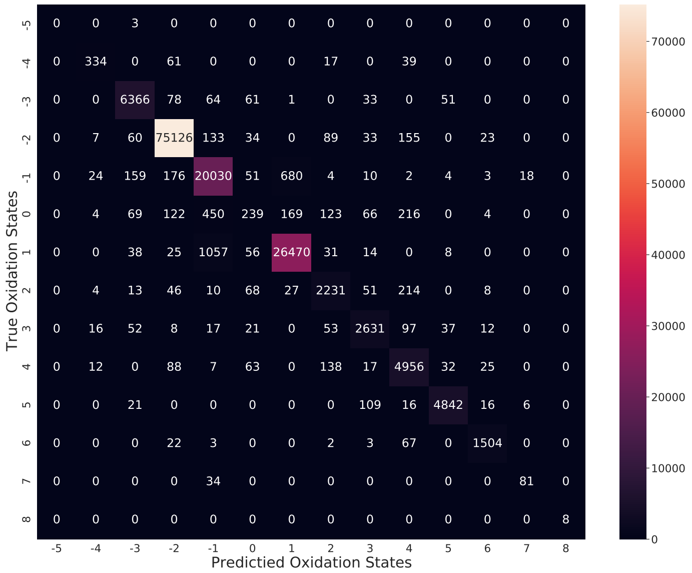

Figure S1: Confusion matrix of non-metal element OS predictions. Overall accuracy: 96.05%. The oxidation states that are easiest to predict for non-metal elements are -2, -1, +1, +4, +3, +5.

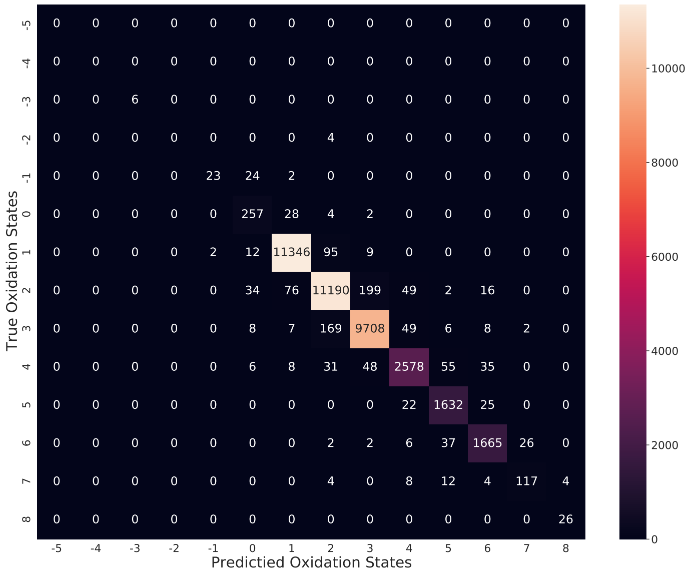

Figure S2: Confusion matrix of metal element OS predictions. Overall accuracy: 97.12%. The oxidation states that are easiest to predict of metal elements are +1, +2, +3.

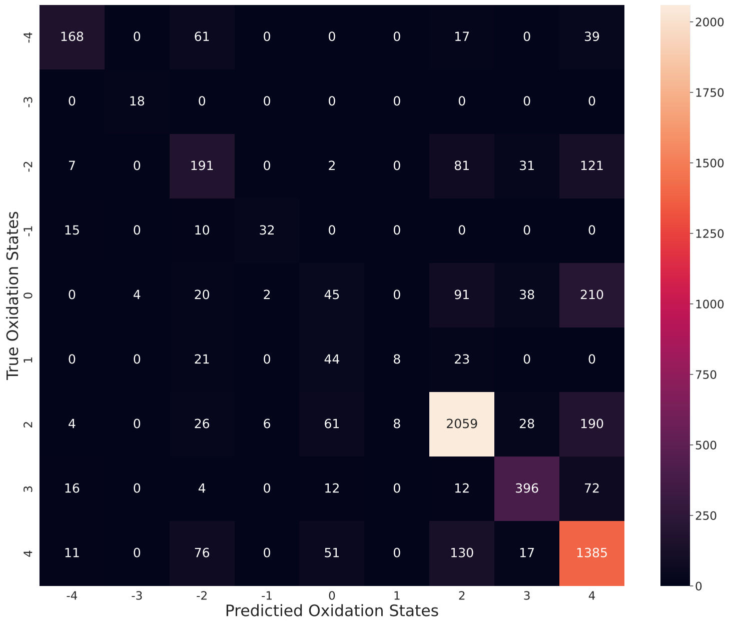

Figure S3: Confusion matrix of the carbon element OS predictions to show the source of error for the carbon element. Overall accuracy: 73.38%.

5 Case studies of predicting complex oxidation states

Table S4: Case studies of complex OS predictions

≥1 transitionMetal + ≥1 non-metal		
	Composition	OS
Ternary	Cr8 P16 O52	**3,5,-2**
	Si2 Ag8 O8	**4,1,-2**
	Cs8 Mn4 Te8	1,2,-2
	Ag20 Sb4 S16	1,3,-2
	Cr4 Ni2 Se8	**3,2,-2**
Quartenary	Rb4 Sm8 Cu4 S16	1,3,1,-2
	Y6 Cu2 Si2 S14	3,1,4,-2
	Mn4 P8 H8 O28	**2,5,1,-2**
	Ta8 H60 N24 Cl28	5,1,-3,-1
	K8 Ti8 P8 O40	1,4,5,-2
>= 5 elements	Mg4 Hg8 H48 Br24 O24	2,2,1,-1,-2
	Na12 Ti6 Fe6 P18 O72	1,4,3,5,-2
	Cs6 Mn2 Cd6 P24 O72	1,3,2,5,-2
	In4 Cu2 Ag2 Te4 Se4	3,1,1,-2,-2
	U4 Ag3 P4 H25 O36	**6,1,5,1,-2**

6 Hypothetical material compositions generated by BLMM and filtered by BERTOS

Top 1000 recommended material compositions by BERTOS

composition	formation_energy_preded(eV)
Cs3CrF7	−6.786247253
Cs3CrF6	−6.019856453
Cs3CrF8	−5.872228622
Lu4OF10	−4.538651466
Tm3O2F5	−4.538619518
Th2F8	−4.532681942
ThF4	−4.532681942
Th3F12	−4.532681942
Tm4O2F8	−4.530905724
LaYF6	−4.486931801
AcF3	−4.485812187
ErF3	−4.474337101
Er3F9	−4.474337101
Er2F6	−4.474337101
YbF2	−4.473279953
Cs2CrF5	−4.470129967
LaEr2F9	−4.469544888
SrTmF5	−4.460558891
LaThF7	−4.459163666
PrTmF6	−4.455236435
TbF3	−4.454846382
Tb4F12	−4.454846382
NdF3	−4.449071884
Nd2F6	−4.449071884
CaTh2F10	−4.446762085
HoF3	−4.446552277
Ho3F9	−4.446552277
BaTm2F8	−4.444099426
HoThF7	−4.440079212
BaEr2F8	−4.434433937
Er5OF13	−4.432486534
Er4OF10	−4.428631783
LaScF6	−4.42856884
Lu3O2F5	−4.428390503
Gd2LuF9	−4.42836237
BaTmF5	−4.424339294
Lu5O3F9	−4.421677113
DyF3	−4.418365002
PrScF6	−4.41412735
Ba2Er3F13	−4.413409233
LaF3	−4.409375191
BaErF5	−4.40564537
SrThF6	−4.404682636
PrF3	−4.399832726
Pr2F6	−4.399832726
TbHo2F9	−4.389440536
SrY4F14	−4.3879776
SrYF5	−4.38557148
CaThF6	−4.383653641
Ba2Lu2F10	−4.382406712
SrLaF5	−4.382071495
SrY2F8	−4.380838394
Y4OF10	−4.369504929

SrYbF4	−4.366775513
Er4O2F8	−4.361657143
Er2OF4	−4.361657143
SmF3	−4.357842445
YF3	−4.347293854
Y2F6	−4.347293854
BaYF5	−4.344537735
BaHoF5	−4.343046188
ThO2	−4.341475487
Th4O8	−4.341475487
Th2O4	−4.341475487
Th7O14	−4.341475487
CaErF5	−4.341230392
KHo3F10	−4.336036682
TmOF	−4.332425117
Y3OF7	−4.330039978
TbHoF6	−4.329440594
ThZrF8	−4.328431129
LuOF	−4.327018738
SrSc2F8	−4.32440567
Ba2HoF7	−4.3237257
BaGd3F11	−4.322098255
SrScF5	−4.320862293
La2OF4	−4.320281506
NaTh2F9	−4.318770409
BaNdF5	−4.318393707
Ba2Nd2F10	−4.318393707
BaGdF5	−4.317327499
Pr3AlF12	−4.315034866
Y2ZrF10	−4.313949585
YbNdF5	−4.313854218
BaSmF5	−4.312891483
NaEr3F10	−4.312729836
CaScF5	−4.312332153
Ca2Sc2F10	−4.312332153
CaSmF5	−4.31150341
Gd4OF10	−4.306436062
BaHfF6	−4.302175999
Y5ClF14	−4.30140686
Sr2YF7	−4.298527718
Er2O2F2	−4.297194004
ErOF	−4.297194004
SrHfF6	−4.293819427
Y6O3F12	−4.293807983
Ce2F6	−4.290774822
CeF3	−4.290774822
Ba2ThF8	−4.287703514
AcSmF6	−4.28660202
Y8O6F12	−4.286550522
KEr2F7	−4.285007477
ScF3	−4.283858299
KLu2F7	−4.282606125
LaZrF7	−4.280694008
ThOF2	−4.280028343
BaDy2F8	−4.279003143
Ba2YF7	−4.278483868

Y3O2F5	−4.278130531
RbEr2F7	−4.276576042
Sr2ScF7	−4.274007797
Sr2LaF7	−4.268908501
Gd3O2F5	−4.268212318
BaScF5	−4.267414093
Ba2Sc2F10	−4.267414093
NaLu3F10	−4.266010284
RbLu2F7	−4.265704632
KHo2F7	−4.264605045
Ca2ScF7	−4.263522625
LiNd3F10	−4.25207901
KY6F19	−4.250665665
Ba2ScF7	−4.248494148
NaEr2F7	−4.248457909
TbOF	−4.247897625
Ho6NpF22	−4.244308472
HoOF	−4.243896008
LiEr2F7	−4.24256134
CeTh2O6	−4.241639137
PrZrF7	−4.241400242
SmZrF7	−4.238841057
Lu2F6	−4.23883152
LuF3	−4.23883152
BaAcF5	−4.237314224
Ho2AlF9	−4.23688221
K2Er3F11	−4.234869957
RbTb2F7	−4.232702255
Dy2AlF9	−4.232624054
ThZr2F12	−4.230744362
DyOF	−4.225504875
Dy2O2F2	−4.225504875
DyZrF7	−4.220570087
CsEr2F7	−4.219665051
YOF	−4.218164444
Y2O2F2	−4.218091965
Gd2MgF8	−4.212934494
CaTh3O7	−4.207875729
Ca3ScF9	−4.207338333
KPr2F7	−4.206991196
KY2F7	−4.205790043
K2Y4F14	−4.205790043
BaCeF5	−4.205186844
CeThO4	−4.204267025
NdAlF6	−4.203757763
HoZrF7	−4.201816559
LaOF	−4.201808929
SrF2	−4.201109886
Sr3F6	−4.201109886
Sr2F4	−4.201109886
ErZrF7	−4.200584412
LaAlF6	−4.196883202
Ba2ZrF8	−4.1967659
CeZrF7	−4.194800854
BaF2	−4.194487095
Ba2F4	−4.194487095

Sr3YF9	−4.190804958
ThOF	−4.190121651
PrAlF6	−4.18884182
NaHo2F7	−4.188398361
SrTh3O7	−4.186162949
Ba3SmF9	−4.186152458
LiDy2F7	−4.185626984
CaZrF6	−4.184970379
Ce2MgF8	−4.182547569
LuPaO4	−4.182151794
YPaO4	−4.182065964
HoAlF6	−4.18186903
GdOF	−4.180844784
Gd2O2F2	−4.180844784
CaF2	−4.179599762
Ca2F4	−4.179599762
Ca3F6	−4.179599762
Ba3Zr2F14	−4.179314613
ThMgF6	−4.177699089
TbAlF6	−4.177511215
YbAlF5	−4.175528526
BaCeF6	−4.17470789
AcOF	−4.173394203
BaEuF4	−4.173036575
CsY2F7	−4.171015263
LuF4	−4.170628548
SmOF	−4.170545578
Ba2CaF6	−4.169711113
SrEuF4	−4.169696808
BaZrF6	−4.169136047
DyPaO4	−4.168826103
Zr2ScF11	−4.168265343
SrCeF6	−4.167639256
SrZrF6	−4.167390823
TmF3	−4.161991119
KThF5	−4.160820007
SmAlF6	−4.159650326
KGd2F7	−4.159254074
BaCaF4	−4.157043457
GdF3	−4.15516901
Gd2F6	−4.15516901
KHf3F13	−4.15387249
ThPaO4	−4.152501583
LiY2F7	−4.151658535
LaPaO4	−4.149893761
CsDy2F7	−4.147538185
GdAlF6	−4.146459579
AmOF2	−4.145533562
NaThF5	−4.143876553
Hf2ThO6	−4.143332005
RbHf3F13	−4.141217232
GdZr2F11	−4.14033699
TbPaO4	−4.139617443
Th2TlF9	−4.13958168
PaF5	−4.138865471
PrZr2F12	−4.138665676

Sr2MgF6	−4.138349533
Th2UF13	−4.13812542
CeZrF8	−4.135380745
PaOF2	−4.134922028
SmPaO4	−4.134225845
HfF4	−4.131919861
Hf2F8	−4.131919861
LiTm2F7	−4.131678581
Eu2AlF9	−4.129302979
CsThF5	−4.128994942
NaHf3F13	−4.12888813
BaZr2F10	−4.128429413
SrZr2F10	−4.127688408
RbThF5	−4.127655983
PrZr5F22	−4.127509594
KHf2F9	−4.127096176
NaY2F7	−4.125955582
Gd2TiF9	−4.125308037
Ca3AlF9	−4.124606133
HfZrF8	−4.123614311
PrOF	−4.120996475
LiThF5	−4.12001276
DyAlF5	−4.118804932
PrPaO4	−4.118695259
CsGd2F7	−4.118637085
TmF4	−4.118196487
Ba3GdF10	−4.117887497
SrCaF4	−4.11773777
Ba2AlF7	−4.11715126
CePaO4	−4.116871834
Sr3AlF9	−4.116252899
K2Gd3F11	−4.113048553
Ba2MgF6	−4.111637115
Ba2ScF8	−4.111274242
CeF4	−4.110645294
Ce2F8	−4.110645294
Ce3F12	−4.110645294
AmZrF8	−4.109612465
Ce2ZrF12	−4.109294891
CeZr2F12	−4.107563019
NdPaO4	−4.107341766
RbHf2F9	−4.104709625
TbZr2F12	−4.103293419
YbCmF5	−4.102480888
Rb2Er2F8	−4.101768494
RbErF4	−4.101768494
Ba3Al2F12	−4.101355553
Ca2AlF7	−4.100431919
NdOF	−4.100406647
Nd4O4F4	−4.100406647
EuAlF6	−4.099006653
K2Er2F8	−4.096999168
KErF4	−4.096999168
KTmF4	−4.096844673
Sr2AlF7	−4.096420288
Tm4TlF13	−4.09618187

Ba3TiF10	−4.095230103
Ba2BeF6	−4.09282589
CaTh2O5	−4.09179306
CaZr3F14	−4.091655731
SmZrF6	−4.090631008
BkTh2O6	−4.089115143
CeAlF6	−4.088780403
ThF3	−4.088383675
Ce3ThO8	−4.086153984
Li2Pr3F11	−4.085495949
ThTiF8	−4.085241318
LiErF4	−4.084342003
Y5PbF17	−4.08381176
SrAlF5	−4.083757401
SrMgF4	−4.083614349
Sr2Mg2F8	−4.083614349
Ba10Cl2F18	−4.082646847
HoPuF7	−4.082163811
Sr2BeF6	−4.081300735
Ba3Mg2F10	−4.080264091
Ba3EuF8	−4.080173016
MgScF5	−4.079357147
K2Lu2F8	−4.079258919
KLuF4	−4.079188347
Dy2ThO5	−4.079056263
NaCe2F7	−4.078036308
CaAlF5	−4.077972889
Ca2Al2F10	−4.077972889
Sr2TiF8	−4.076644421
Er8Hf3O18	−4.076355934
Gd2PaO6	−4.075930595
Tm2HfO5	−4.075503826
Ba2GdF8	−4.075079918
BaAlF5	−4.074412823
Ba2Al2F10	−4.074412823
Ba3Al3F15	−4.074412823
Er6UO12	−4.07108736
CsSc2F7	−4.071071148
LaBeF5	−4.070886135
Ca2TiF8	−4.069008827
La3LuO6	−4.068767548
ScOF	−4.068746567
Ba2TiF8	−4.068396568
NaSc2F7	−4.067670822
RbLuF4	−4.067507744
ZrF4	−4.067088127
Zr2F8	−4.067088127
Zr3F12	−4.067088127
LaEr5O9	−4.066586494
Tm4O6	−4.064805984
Tm2O3	−4.064805984
BaTmF6	−4.064612389
AcF2	−4.064072609
CsYb2F5	−4.06370163
NaLuF4	−4.062561989
KHoF4	−4.062361717

BaTh2O5	−4.060285568
Er4Hf2O10	−4.059690475
CeOF	−4.059506893
Ca2BeF6	−4.059294701
Th4PoO10	−4.058458328
ScPaO4	−4.058246613
KSc2F7	−4.057766438
RbHoF4	−4.057738781
La2ThO5	−4.056145668
Ca2MgF6	−4.054861069
Dy2Hf3O9	−4.054292679
BaAl2F8	−4.054280758
CsErF4	−4.053524971
Ba3Er2F14	−4.052350998
Sr2AlF8	−4.051891327
YbMgF4	−4.051839828
NaErF4	−4.051551819
RbTmF4	−4.050311089
PrPuF7	−4.044788361
RbTbF4	−4.044387341
TbMgF5	−4.043642044
ErMgF5	−4.042970657
Ho2HfO5	−4.042778492
Ce4LuO9	−4.042086601
Y2PaO6	−4.041396141
PaF6	−4.040874958
CaMgF4	−4.040514946
ThU2F12	−4.040272713
YbUF7	−4.039618492
EuF2	−4.03862524
Eu2F4	−4.03862524
Lu2O3	−4.037151337
Lu8O12	−4.037151337
KLaF4	−4.035759449
Ba2EuF7	−4.035627365
CaTiF6	−4.034531116
EuTiF6	−4.034454823
Tm2ZrO5	−4.033969402
AmF4	−4.033653736
KTbF4	−4.032445431
La2BF9	−4.03083992
RbLaF4	−4.030204773
Ba3Ti2F14	−4.026776314
Ba7Cl2F12	−4.026159286
CsLaF4	−4.025967598
BaNpF6	−4.025859833
CsLuF4	−4.025128365
Li2Lu2F8	−4.025117874
SrUF6	−4.024926186
BaMgF4	−4.024667263
Ba2Mg2F8	−4.024667263
NaTmF4	−4.024656296
LuScO3	−4.024190903
Th2TiO6	−4.022697926
Er2Hf2O7	−4.021972179
KYF4	−4.021393776

K2Y2F8	−4.021393776
K3Th2F11	−4.02016449
Li2Dy2F8	−4.019582272
LiDyF4	−4.019582272
Th2SiO6	−4.019172668
Y3O4F	−4.019066811
KNdF4	−4.018717766
CaAl2F8	−4.018482208
LiTbF4	−4.018092155
Ba2TbF8	−4.017904758
La2Hf3O9	−4.017622948
SrAl2F8	−4.017306328
TmLuO3	−4.016262054
HoTiF6	−4.015938759
Dy2Hf2O7	−4.015523434
NaHoF4	−4.01522541
Na2Ho2F8	−4.01522541
LiTmF4	−4.015050888
Nd6O7F4	−4.014970779
Rb2Y2F8	−4.014668465
RbYF4	−4.014668465
Eu2BeF7	−4.013708115
RbNdF4	−4.012892723
TbTiF6	−4.012829781
SrTiF6	−4.012724876
BkThF6	−4.010547638
Cs2CrF6	−4.008408546
Y6UO12	−4.006516933
PrZrF8	−4.006132603
KDyF4	−4.005791187
PaF4	−4.004402161
Ba2TiF7	−4.004188538
AcAmF6	−4.003125191
CsHoF4	−4.001977921
CaUF7	−4.001787186
Ca2U2F14	−4.001787186
LaLuO3	−3.998473883
PuPaO4	−3.998317957
CfSc5O9	−3.997891903
Pr2SF4	−3.997578859
Ba3ClF5	−3.997068405
Er2O3	−3.997007608
Er4O6	−3.997007608
ErLuO3	−3.996432781
DyLuO3	−3.996176958
Ho2ZrO5	−3.995995522
Y3PbF11	−3.995750666
Gd2ThO5	−3.994701385
Dy2HfO5	−3.993907452
HoLuO3	−3.993804932
LiHoF4	−3.993438005
La2Hf2O7	−3.993071318
LiYF4	−3.992584467
La3ErO6	−3.992555618
CsNdF4	−3.990710735
SrMg2F6	−3.990532637

CaUF6	−3.989569426
Y3UO7	−3.989440203
KSmF4	−3.989100218
Ba2SiF8	−3.988746643
NaLaF4	−3.988582134
La4UO9	−3.988389254
CeEr4O8	−3.988114834
CsTbF4	−3.987507343
KPrF4	−3.987312317
NaSmF4	−3.985390663
SrUF7	−3.984490633
CsYF4	−3.984406948
CaU2F10	−3.984314919
PuF3	−3.982479811
Sm2Hf2O7	−3.982316971
Gd2Sc4O9	−3.981632471
SrHf3O7	−3.981467247
RbPrF4	−3.981390953
La3UO7	−3.981289148
TbNpF8	−3.980888844
ThZr2O6	−3.980735064
Th2O3	−3.980697632
Ba4Mg6F20	−3.980134487
Ba2Mg3F10	−3.980134487
BaUF6	−3.978858232
KGdF4	−3.978217363
NaNdF4	−3.97784543
CeY6O11	−3.977183104
Ba3TaF11	−3.977061987
CaHf4O9	−3.976824999
KYbF3	−3.976506233
SrSc6O10	−3.976321459
HoTmO3	−3.976121902
SrBeF4	−3.97602582
Yb2F5	−3.975758553
CfAlF6	−3.975150347
Ba3Ti3F18	−3.974650145
Ba2Ti2F12	−3.974650145
BaTiF6	−3.974650145
CaU2F12	−3.974611044
YLuO3	−3.9745543
EuHfF7	−3.974365234
LaTmO3	−3.974125147
CfPaO4	−3.973865986
PaO2	−3.973798275
Pa2O4	−3.973798275
Er2ZrO5	−3.973560572
TbZrF8	−3.973423481
CsZr2F9	−3.973205328
SrTm4O7	−3.973033428
RbHfF5	−3.972944498
Rb3Hf3F15	−3.972944498
Rb2Hf2F10	−3.972944498
KHfF5	−3.972760677
BaPaF8	−3.972542286
GdU2F13	−3.972445726

DyScO3	−3.972443819
RbZr2F9	−3.972401142
LiGdF4	−3.9719522
BaBeF4	−3.971497059
Ba2Be2F8	−3.971497059
NaYF4	−3.971271515
CsHfF5	−3.971121788
Cs2Hf2F10	−3.971121788
HfO2	−3.970953465
Hf4O8	−3.970953465
Hf2O4	−3.970953465
Hf6O12	−3.970953465
Gd2EuF9	−3.970173836
Tb2ZrO5	−3.969937086
BaGdF6	−3.969935179
Ca2SiF8	−3.969781876
Hf3VF14	−3.969767332
YTaF8	−3.96900034
CaEr8O13	−3.968161821
LiCa2F5	−3.96803093
Tm2O2F4	−3.967924595
CaHf3O7	−3.967892408
LuBeF5	−3.967798948
YbY3F8	−3.96720314
UPaO5	−3.966614246
SmY5O9	−3.966320992
LaY3O6	−3.963053226
SmBeF5	−3.962896824
NaPrF4	−3.961989164
Ba4Ta2F18	−3.961243629
Ba2TaF9	−3.961243629
NaHfF5	−3.960839272
YScO3	−3.960505962
LiHfF5	−3.959972382
TbTmO3	−3.958104372
La4Hf2O10	−3.958006382
La2HfO5	−3.958006382
BkPaO4	−3.957753658
NaEu2F6	−3.956979513
BaMg2F6	−3.956091404
Ba3Mg6F18	−3.956091404
Ba2Mg4F12	−3.956091404
UF4	−3.955328226
U3F12	−3.955328226
U2F8	−3.955328226
SmClF2	−3.954778194
YbBeF4	−3.954603434
Dy4ZrO8	−3.951759338
SrLu4O7	−3.951238632
Nd2ThO5	−3.950950384
EuAlF5	−3.950551748
BaPuF6	−3.950229168
CeF2	−3.94973278
TbHo3O6	−3.948726654
LiSmF4	−3.948374748
CaBeF4	−3.947942019

ErScO3	−3.947625399
CaTaF7	−3.946530581
HoScO3	−3.94581604
Ca2AlF8	−3.945786476
SrPuF6	−3.944443226
KCeF5	−3.944064856
SrY8O13	−3.943619013
Gd3ScO6	−3.943567514
Gd2HfO5	−3.942780018
Ce5ZrO12	−3.942285299
SrHf2O5	−3.94181776
Ba3Ti2F12	−3.941714048
DyTmO3	−3.941419601
BaU2F10	−3.940893888
Zr2UF12	−3.94086194
KEu3F9	−3.940620184
Ba3Ta2F16	−3.940342188
Er4TiO8	−3.94011569
LaErO3	−3.939831257
Cs2Zr3F14	−3.939676762
BaHf3O7	−3.939386129
CsEu3F9	−3.938849449
BaUF7	−3.938765049
CaY4O7	−3.938592911
Dy2ZrO5	−3.938372612
Lu2Zr3O9	−3.937586308
DyHoO3	−3.936909676
GdYO3	−3.936896563
GdScO3	−3.936368465
Gd3Sc3O9	−3.936368465
Gd2Sc2O6	−3.936368465
KPaF6	−3.936228752
GdLuO3	−3.935838938
TmTiF6	−3.935361147
RbYbF3	−3.935252666
TbScO3	−3.935095787
NdSc5O9	−3.934554815
NaCeF5	−3.934113026
Y2Er2O6	−3.933290958
CaHf2O5	−3.933196306
Ca2Hf4O10	−3.933196306
SrMg3F8	−3.933195114
YTiF6	−3.933167219
ThVF7	−3.931904793
NaGdF4	−3.931534052
BaU3F14	−3.930975437
SrTaF7	−3.930519581
SmScO3	−3.930409908
Sm2Sc2O6	−3.930409908
Nd2HfO5	−3.930255651
K2ThF6	−3.9298985
RbCeF5	−3.928171873
LaAlF5	−3.927558422
LaHoO3	−3.927205563
YHoO3	−3.926771402
CsCeF5	−3.926264286

Ba3Ti4F22	−3.92620492
TbDyO3	−3.926163912
Pr2HfO5	−3.926158905
CfF3	−3.925917387
KCeF4	−3.925480604
K2Ce2F8	−3.925480604
SmSc3O6	−3.923517942
GdTmO3	−3.923139334
CePuF8	−3.922807217
KU3F13	−3.922387362
PuThO4	−3.921397686
CaTiF5	−3.921141148
BaSc6O10	−3.921061754
Ba2DyF8	−3.920909882
RbGdF4	−3.920048475
DyYO3	−3.91970253
CsU3F13	−3.919485331
CsYbF3	−3.919182301
NaDyF4	−3.918666601
Ca2TbF8	−3.918600559
UAlF7	−3.918360949
Li2Pr2F8	−3.918358326
Ce3ZrO8	−3.918244362
Y2UO6	−3.915689945
Tm3ClO4	−3.91510272
BaEr6O10	−3.914818048
Zr2UF13	−3.914527178
AmOF3	−3.914085627
BaMg3F8	−3.913954496
Gd2Hf2O7	−3.912679434
Ho5TaO10	−3.912414074
YUO4	−3.912060261
Y2U2O8	−3.912060261
Y2PbF8	−3.911138773
Y2ZrO5	−3.911123037
NdScO3	−3.910733223
Nd2Sc2O6	−3.910733223
LiU3F13	−3.910445452
CeO2	−3.909799337
Ce4O8	−3.909799337
Ce2O4	−3.909799337
Ce10O20	−3.909799337
Ce3O6	−3.909799337
KScF4	−3.909413099
NdSc3O6	−3.909043789
La2O3	−3.908939123
La4O6	−3.908939123
LiZr2F9	−3.908349276
LaF4	−3.908234119
EuMgF5	−3.908163786
RbPaF6	−3.908007145
Ce2OF6	−3.907115698
TbYO3	−3.906951904
BaLu4O7	−3.906234741
ErOF2	−3.906198263
Er3AlO6	−3.905852318

Tb2ZnF8	−3.905344009
Ba2NbF9	−3.903748274
BaHf2O5	−3.903716326
BaTm4O7	−3.903196096
Sr3BF9	−3.903009653
CsGdF4	−3.902306557
NdLuO3	−3.90143919
Sr2Hf3O8	−3.899674654
Y2O3	−3.899650812
Y6O9	−3.899650812
Y4O6	−3.899650812
Y8O12	−3.899650812
Y12O18	−3.899650812
PrF2	−3.899086237
CaUF8	−3.89789176
RbU3F13	−3.897682428
GdDyO3	−3.897519827
DyZrF8	−3.897320986
CeSc2O5	−3.897313833
SrTiF5	−3.896959066
NaZr2F9	−3.896894217
AlF3	−3.89687109
Al2F6	−3.89687109
HoTaF8	−3.89649272
NdBF6	−3.896488667
BaPrF4	−3.896294832
NaScF4	−3.895940065
Na3Sc3F12	−3.895940065
Na2Sc2F8	−3.895933628
LaScO3	−3.89534831
La2Sc2O6	−3.89534831
RbU4F21	−3.895327091
SmBF6	−3.895288944
Hf2TlF9	−3.895126343
LaTa2F13	−3.894266367
LaUO4	−3.894155741
La2U2O8	−3.894155741
Hf2MnF10	−3.894045591
Ba2AlF6	−3.893369198
U2F10	−3.892789125
UF5	−3.892789125
Cs2ThF6	−3.892372131
LiScF4	−3.892340183
Li2Sc2F8	−3.892340183
Lu3CuF11	−3.892089367
Ce2ZrO6	−3.892060041
BaSiF6	−3.892007589
SmTmO3	−3.891228199
RbScF4	−3.890647173
Rb2Sc2F8	−3.890647173
Er4BeO7	−3.890621901
Sc2O3	−3.89016223
Sc4O6	−3.89016223
Sc6O9	−3.89016223
KPaF5	−3.889205456
PuPaO5	−3.889129639

Ce3Zr2O10	−3.889091492
BaTaF7	−3.888895988
CsScF4	−3.888829947
Cs2Sc2F8	−3.888829947
KZr5F21	−3.88851285
Ho2Zr2O7	−3.887775898
Nd4AgF13	−3.887429714
Ca2Hf3O8	−3.887236595
NaCeF4	−3.886569738
Zr2TiF10	−3.885798216
BkThO4	−3.885032415
LaTbO3	−3.88498807
SrSiF6	−3.884242058
La2Sc4O9	−3.884129763
Ho2PbF8	−3.884059429
Sm2Y2O6	−3.883264303
SmYO3	−3.883264303
Er3TaO7	−3.88297677
Rb2ThF6	−3.88263464
Ce3ZrO7	−3.88228488
Sm3UO7	−3.881890535
LaDyO3	−3.88113451
ThTiO4	−3.880225658
LiU2F9	−3.88008523
Zr4IF19	−3.879184008
PrSc3O6	−3.879158497
Na4Hf3F16	−3.878847122
Ba2Mg8F20	−3.8788414
Dy2BF9	−3.878667116
Sr2Sc6O11	−3.878422499
CsCeF4	−3.877542734
Lu4Si2O10	−3.877373219
Lu2SiO5	−3.877373219
LuUO4	−3.876790762
Cs2U4F18	−3.876717329
CsU2F9	−3.876717329
SmErO3	−3.876227617
PrScO3	−3.87601161
Pr3Sc3O9	−3.87601161
LuAlF5	−3.875945807
EuPaO4	−3.875561237
EuPaO4	−3.875561237
Na2Zr3F14	−3.875551224
YbThO3	−3.875174284
Ho3TaO7	−3.873947859
AcF4	−3.873799562
KZr2F9	−3.873456717
UO2F	−3.873129606
NaU3F16	−3.872771978
BaY6O10	−3.872710228
Nd3ScO6	−3.872585535
LiCeF5	−3.872513294
CsBa2F5	−3.871919394
BaTbF6	−3.871601105
PuAlF7	−3.870946169
CeUO4	−3.869502306

Na4Ho3F13	−3.869100571
NdThO4	−3.86905551
PrTmO3	−3.868521214
Rb3Zr3F15	−3.868383884
RbZrF5	−3.868383884
Rb2Zr2F10	−3.868383884
HfZr2O6	−3.868074894
BaSc4O7	−3.867880344
CeGd2O5	−3.86775279
Tb2Zr2O7	−3.865983486
Y3AlO6	−3.865933657
Hf2UO6	−3.865755558
SrY4O7	−3.864356518
K2U3F14	−3.862414122
NaU2F11	−3.861394167
SmAl2F8	−3.861116409
NaU2F9	−3.860802174
Dy3TaO7	−3.860769987
Ce2Zr2O8	−3.860695124
CeZrO4	−3.860695124
Ce3Zr3O12	−3.860695124
Cs3Zr3F15	−3.859544516
CsZrF5	−3.859544516
Cs2Zr2F10	−3.859544516
Ca3Hf4O11	−3.859415054
Tm2SiO5	−3.859366894
SmGdO3	−3.859033585
Tb3TaO7	−3.858229637
Er2Zr2O7	−3.858149767
CeScO3	−3.857439995
Er2Zr3O9	−3.857149839
Tb2CdF8	−3.857035875
KU2F11	−3.856789112
PrUO4	−3.856770039
ThNp2F14	−3.85661149
Na2ThF6	−3.856070995
HfUO4	−3.855912685
LaYO3	−3.855646372
CeBeF6	−3.855170965
NdTmO3	−3.853690147
CaTm2O4	−3.853420734
SrSmF4	−3.852856874
La3TaO7	−3.852784157
BaLaF4	−3.852413654
LiCeF4	−3.851648092
NdHoO3	−3.851085901
AmF3	−3.851001978
DyUO4	−3.850905895
CeTmO3	−3.850883722
BaTiF5	−3.850869179
Dy3AlO6	−3.850812197
Gd2ZrO5	−3.85040617
TbHoO3	−3.850380182
LaGdO3	−3.850368023
BaTi2F9	−3.850194931
Y2Zr2O7	−3.850127935

NdYO3	−3.85002327
CeBeF5	−3.849918842
NdUO4	−3.84890604
LiZrF5	−3.848706007
Li2Zr2F10	−3.848706007
CaLu2O4	−3.848416328
NaYbF3	−3.847837925
Ba3Ho3F18	−3.847299576
BaHoF6	−3.847299576
Ba2NbF8	−3.84631896
ZrTiF8	−3.845727205
Gd4SiO8	−3.844508648
Pr2Zr2O7	−3.844108343
TbF2	−3.843782425
Dy2Zr2O7	−3.843649626
Ca2BF7	−3.843381166
Np2F8	−3.842690706
NpF4	−3.842690706
Sm2ZrO5	−3.842666626
KZrF5	−3.842362881
K2Zr2F10	−3.842362881
K3Zr3F15	−3.842362881
PrBF6	−3.842349768
U3OF13	−3.842239141
Er4Al2O9	−3.842167139
BaThF8	−3.841967106
La2TiO5	−3.841740608
La4Ti2O10	−3.841740608
Y4Ti2O10	−3.841731071
Y2TiO5	−3.841731071
Sm2Zr2O7	−3.841445446
SrTm2O4	−3.83963728
Sr2Tm4O8	−3.83963728
CmOF	−3.839493513
Sr3VF10	−3.839368343
YF4	−3.839230776
YbZrF7	−3.837999582
Y3TaO7	−3.837685108
TbNd5O9	−3.83694458
ScUO4	−3.836723328
LaVF6	−3.836299896
BaBe2F6	−3.836184978
PuF4	−3.835775614
TbSmO3	−3.835399866
Li3Nd2F9	−3.835178137
K2ErF5	−3.834710836
PrLuO3	−3.834599733
BaY4O7	−3.834504366
Gd2TiO5	−3.83439064
Zr3VF14	−3.833183765
CeErO3	−3.833114386
Zr3MnF14	−3.832733393
Mg2BeF6	−3.832675457
Ce3Zr4O14	−3.832588196
Th2Ta2O9	−3.830219269
HoUO4	−3.829547882

Ho2TiO5	−3.829313517
BaSmF4	−3.8288486
NaZrF5	−3.828633308
Na2Zr2F10	−3.828633308
Ho2SiO5	−3.828163385
Gd3TaO7	−3.827930212
PrAlF5	−3.827482939
CmF3	−3.827294827
Gd3AlO6	−3.827104568
SrEr4O7	−3.827076674
K2PaF7	−3.826327085
Sr2Hf2O6	−3.825815201
Sr3Hf3O9	−3.825815201
SrHfO3	−3.825815201
La2Zr2O7	−3.825736523
AmOF	−3.825441837
Dy2SiO5	−3.825042009
Ce2Zr3O10	−3.824987888
Pr2Zr3O9	−3.82491827
K2TmF5	−3.824710846
ThSiO4	−3.824430466
CeZr2O6	−3.824424267
Ce2Zr4O12	−3.824424267
La2SiO5	−3.824226379
AcHoO3	−3.824111462
U4TlF17	−3.823364019
Cs2Ti5F22	−3.822115183
BaCe4O9	−3.82126236
Y2Zr3O9	−3.820122957
LaSmO3	−3.819991827
Er7GaO12	−3.819559574
SmUO4	−3.819553614
Sr3VF9	−3.819257498
Lu2Ti2O7	−3.819142342
CeZr3O8	−3.818742752
CeDy4O9	−3.818337917
Nd2ZrO5	−3.817111254
La2Zr3O9	−3.817110538
Ba3Cl2F4	−3.816862583
TbNdO3	−3.816772699
Tm2TiO5	−3.81658864
Tm4Ti2O10	−3.81658864
Cs2LuF5	−3.816576719
Ce2TiO6	−3.815433264
Pr2ZrO5	−3.815412283
Ba2Ti5F21	−3.815376759
Rb2ErF5	−3.814884424
CaBe2F6	−3.814664125
K2LuF5	−3.814659119
Tb2TiO5	−3.814434052
K2LaF5	−3.814233541
CeZr4O10	−3.814175129
Dy2TiO5	−3.812735796
Rb2LuF5	−3.811721563
TmUO4	−3.811430216
SrErF6	−3.810247421

Zr2MnF10	−3.810170174
PuOF	−3.809472322
La4Ti3O12	−3.809059381
Er2TiO5	−3.808876038
RbAl2F7	−3.807883739
Tb2O3	−3.807523489
Tb4O6	−3.807523489
Y2SiO5	−3.807328701
CsAl3F10	−3.807319403
Nd2Zr2O7	−3.807163954
AcErO3	−3.806783199
SrTi2F8	−3.806652784
NdF4	−3.806259632
SrLu2O4	−3.80597496
TbGdO3	−3.805543661
AcLuO3	−3.805460691
CeYO3	−3.805301189
K5Y3F14	−3.804858446
CsAl2F7	−3.804744005
Cs2Al4F14	−3.804744005
Tb2SiO5	−3.804600954
CsTi4F17	−3.804323435
LaF2	−3.804077387
Ce2Zr2O7	−3.80355835
K5Zr4F21	−3.803414106
Er2SiO5	−3.803329945
Er2UO6	−3.803126574
PrYO3	−3.802994251
Sm2Zr3O9	−3.802596092
LaOF2	−3.802201271
EuY3O6	−3.801918983
CaThO3	−3.801113367
Ca2Ho4O8	−3.800702333
CaHo2O4	−3.800702333
Ba2Y6O11	−3.80063343
La3AlO6	−3.800589323
Nd4SiO8	−3.800578117
Ba3IF5	−3.799977779
Yb2CdF6	−3.799782753
Sc2TiO5	−3.799678802
LiLuF5	−3.799258947
ThPbF6	−3.79886508
BaScF4	−3.798820496
SmF4	−3.798787832
CaAlF4	−3.798273563
Ho2PaO6	−3.798186302
Rb3Zr2F11	−3.797622919
La2Ti2O7	−3.797019243
La4Ti4O14	−3.797019243
CaSc2O4	−3.796969414
